# A randomized 3-way crossover study indicates that high-protein feeding induces de novo lipogenesis in healthy humans

**DOI:** 10.1172/jci.insight.124819

**Published:** 2019-05-30

**Authors:** Evelina Charidemou, Tom Ashmore, Xuefei Li, Ben D. McNally, James A. West, Sonia Liggi, Matthew Harvey, Elise Orford, Julian L. Griffin

**Affiliations:** 1 Department of Biochemistry and Cambridge Systems Biology Centre, University of Cambridge, Cambridge, United Kingdom.; 2 Division of Gastroenterology and Hepatology, Department of Medicine, Addenbrooke’s Hospital, University of Cambridge, Cambridge, United Kingdom.; 3 Medical Research Council — Elsie Widdowson Laboratory, Cambridge, United Kingdom.; 4 Computational and Systems Medicine, Surgery and Cancer, Imperial College London, London, United Kingdom.

**Keywords:** Metabolism, Amino acid metabolism, Diabetes, Obesity

## Abstract

**BACKGROUND:**

Dietary changes have led to the growing prevalence of type 2 diabetes and nonalcoholic fatty liver disease. A hallmark of both disorders is hepatic lipid accumulation, derived in part from increased de novo lipogenesis. Despite the popularity of high-protein diets for weight loss, the effect of dietary protein on de novo lipogenesis is poorly studied. We aimed to characterize the effect of dietary protein on de novo lipid synthesis.

**METHODS:**

We use a 3-way crossover interventional study in healthy males to determine the effect of high-protein feeding on de novo lipogenesis, combined with in vitro models to determine the lipogenic effects of specific amino acids. The primary outcome was a change in de novo lipogenesis–associated triglycerides in response to protein feeding.

**RESULTS:**

We demonstrate that high-protein feeding, rich in glutamate, increases de novo lipogenesis–associated triglycerides in plasma (1.5-fold compared with control; *P* < 0.0001) and liver-derived very low-density lipoprotein particles (1.8-fold; *P* < 0.0001) in samples from human subjects (*n* = 9 per group). In hepatocytes, we show that glutamate-derived carbon is incorporated into triglycerides via palmitate. In addition, supplementation with glutamate, glutamine, and leucine, but not lysine, increased triglyceride synthesis and decreased glucose uptake. Glutamate, glutamine, and leucine increased activation of protein kinase B, suggesting that induction of de novo lipogenesis occurs via the insulin signaling cascade.

**CONCLUSION:**

These findings provide mechanistic insight into how select amino acids induce de novo lipogenesis and insulin resistance, suggesting that high-protein feeding to tackle diabetes and obesity requires greater consideration.

**FUNDING:**

The research was supported by UK Medical Research Council grants MR/P011705/1, MC_UP_A090_1006 and MR/P01836X/1. JLG is supported by the Imperial Biomedical Research Centre, National Institute for Health Research (NIHR).

## Abstract 2

A subset of amino acids may induce de novo lipogenesis in humans, suggesting that use of high-protein diets to tackle diabetes requires greater consideration.

## Introduction

Recent rapid changes in diet have coincided with modernization, urbanization, economic development, and increased population wealth, and have had a profound effect on body composition and health status (1). The transition from traditional diets, high in cereal and fiber, to Western-style diets, high in sugars, fat, and animal-derived products, is thought to be a key contributor to the obesity epidemic and increased incidences of metabolic syndrome, type 2 diabetes mellitus (T2DM), and nonalcoholic fatty liver disease (NAFLD) (2).

Both NAFLD and T2DM (3) have been associated with increased hepatic lipid production via de novo lipogenesis (DNL). This pathway synthesizes new fat, utilizing acetyl-coenzyme A (CoA) as a carbon source, derived from a number of metabolic reactions, including glycolysis or the deamination of amino acids. Fatty-acyl chains can then be incorporated into a variety of lipid species, including triacylglycerols (TAGs) and phospholipids. The pathway commences by carboxylation of acetyl-CoA to malonyl-CoA by acetyl-CoA carboxylase (ACC). Malonyl-CoA is then transferred to a complex multifunctional enzyme, fatty acid synthase (FAS). Multiple rounds of activation of acetyl-CoA to malonyl-CoA, transferral to FAS, and addition to the lengthening carbon chain generate palmitate (4).

DNL is regulated at a transcriptional level by both glucose and insulin. It has previously been established that hyperinsulinemia and high carbohydrate diets “prime” DNL by providing a large substrate pool (5, 6). Glucose also stimulates the expression of anabolic DNL genes, including those encoding ATP-citrate lyase (ACL), ACC, and FAS (*ACLY*, *ACACA*, and *FASN*, respectively) by the action of the transcription factor carbohydrate response element–binding protein (ChREBP) (7). In turn, insulin activates the same genes, via protein kinase B/AKT2 (8), by the action of SREBP-1c, one of 2 transcripts produced from the sterol response element–binding protein gene. Once fatty acids (FAs) are synthesized, they are desaturated via the action of stearoyl-CoA desaturase 1 (SCD1) and/or elongated by elongation of very long FAs protein 6 (ELOVL6). Finally, FAs may be incorporated into TAGs and packaged into VLDLs for export.

Accumulation of a subset of shorter-chain TAGs (scTAGs) and VLDL-associated scTAGs in the liver and blood, respectively, has been associated with DNL and hepatic steatosis (9). In healthy humans, the amount of de novo produced TAG incorporated in VLDL is small (below 5%) (10). However, in hyperinsulinemic individuals with NAFLD, de novo produced TAG content increases to 26% (4). Furthermore, in healthy human subjects, feeding with a high-carbohydrate meal is sufficient to increase DNL-derived TAG content to 23% (10). While numerous studies have shown that high-carbohydrate feeding increases DNL (11), studies of the effect of high-protein feeding in relation to DNL in humans are limited.

The branched-chain amino acids (BCAAs) leucine, isoleucine, and valine are known to correlate with insulin resistance and T2DM in humans (12). Studies have demonstrated that deprivation of BCAAs from the diet while maintaining levels of other amino acids is sufficient to improve glycemic control (13). Increased circulating BCAAs are sensed by mTOR, a protein kinase that is a critical node of the insulin signaling cascade, and subsequently promote DNL (14). Hyperactivation of the mTOR pathway by excessive amino acid concentrations leads to phosphorylation of multiple serine residues within, and subsequent inactivation of, insulin receptor substrate 1 (IRS-1) (15). This results in a weakened response to insulin, progressing to persistent insulin resistance. Despite this, high-protein/low-carbohydrate diets are popular regimes for weight loss in humans, and dietary protein has been shown to decrease hepatic lipid accumulation in rodents (16). At present, the precise amino acid components that are responsible for these effects, and the extent to which amino acids are able to induce DNL and VLDL secretion, are not known.

The primary objective of this study was to characterize the effect of dietary amino acids on DNL. We demonstrate that high-protein feeding, using a soy protein intervention rich in glutamate, results in increased levels of scTAGs and VLDL-associated scTAGs, which have been associated with DNL (9), in plasma of human male subjects. Only males were recruited due to the large difference between the lipid profiles of men and premenopausal women arising from different sex hormones, which are important regulators of lipid metabolism (17). We also determine that glutamate induces DNL and is itself incorporated in FAs via the TCA cycle in hepatocytes, most likely via reductive carboxylation. In addition, supplementation with leucine and glutamine in hepatocytes resulted in a significant increase in scTAGs as well as a decrease in glucose uptake. This may provide insight into the negative effects of BCAAs on insulin sensitivity and metabolic health, and was recapitulated with both glutamine and glutamate but not lysine supplementation.

## Results

### scTAGs are major discriminant variables between high-protein- and control-fed healthy male subjects.

**Figure 1:**
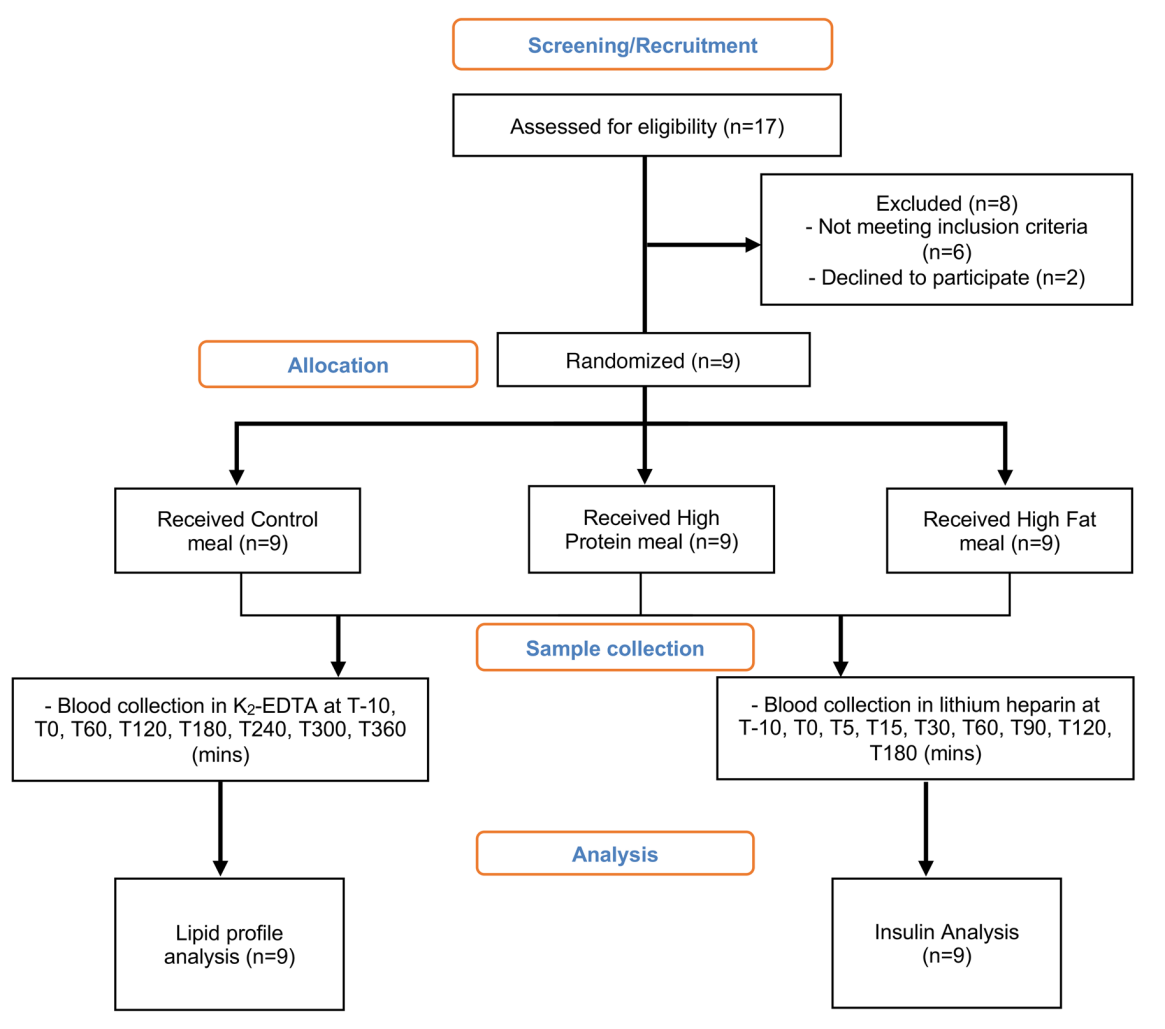
Study design flowchart. Seventeen males were screened to examine whether they met the inclusion criteria of the study. Six of them did not meet the criteria, and another 2 declined to participate. Nine volunteers participated in a randomized 3-way-crossover study. The same 9 volunteers attended the Medical Research Council – Elsie Widdowson Laboratory on 3 different occasions and received one of the 3 isoenergetic meals (control [C], high-protein [HP], and high-fat [HF]) in a randomized order each time. Blood samples were collected at different time points for lipid profile and hormonal analysis. The study was ended once all participants consumed the 3 meals.

**Figure 2:**
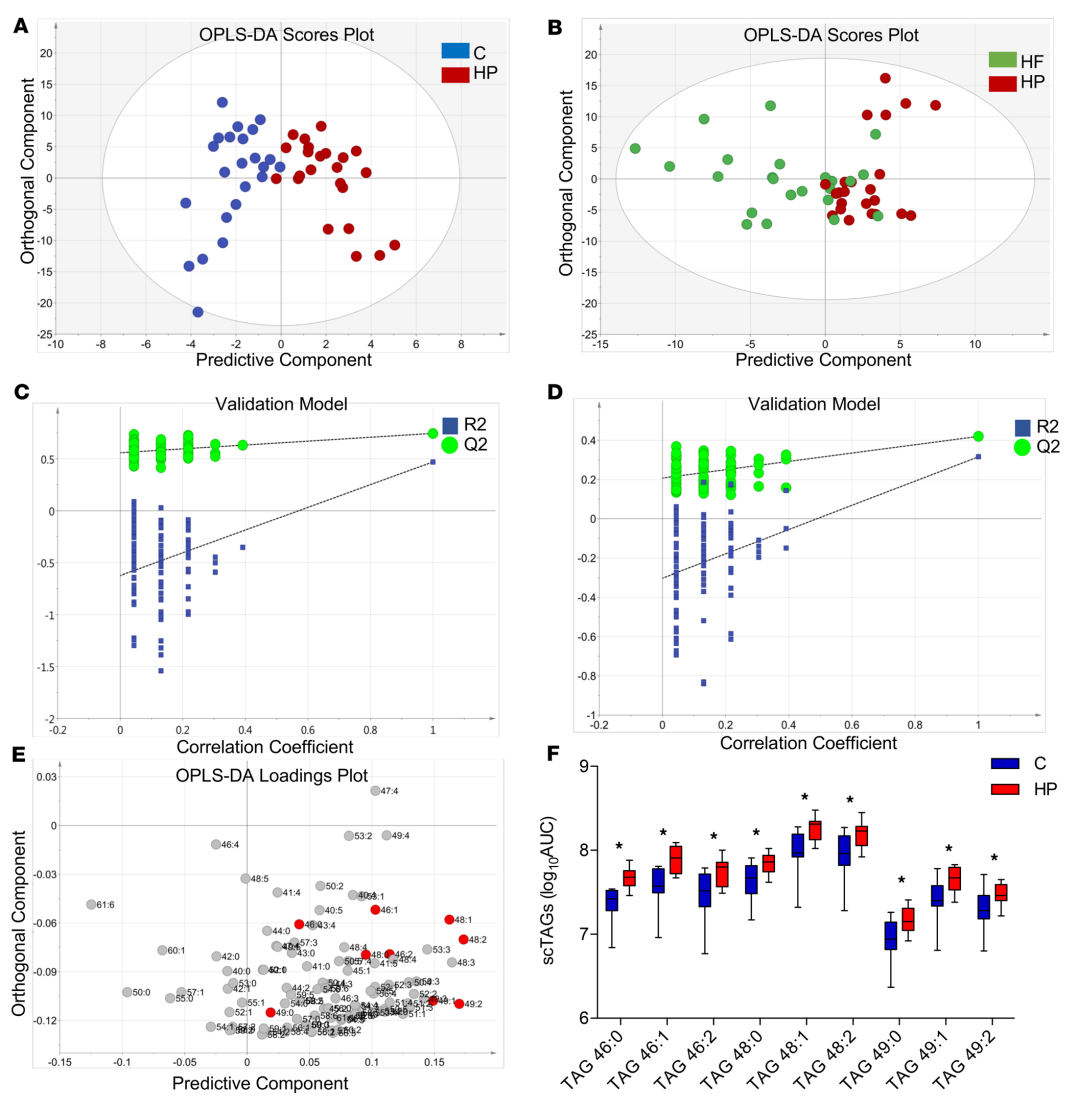
Multivariate data analysis of healthy human subjects fed control, high-protein, and high-fat meal. (**A**) OPLS-DA scores plot discriminating TAG profiles in plasma of individuals fed control [C] and high-protein [HP] meals after 3, 4, and 5 hours. Each blue circle represents a single C-fed individual, while red represents HP-fed individuals (*R^2^X* = 0.84, *Q^2^* = 0.47). (**B**) OPLS-DA scores plot discriminating TAG profiles in plasma of HF-and HP-fed individuals after 3, 4, and 5 hours. Each green circle represents a single HF-fed individual, while red represents HP-fed individuals (*R^2^X* = 0.54, *Q^2^* = 0.32). (**C**) Cross-validation of the model in **A** acquired through 100 permutation tests; *y*-axis intercepts: *R^2^* = (0.0, 0.56), *Q^2^* = (0.0, –0.63). *n* = 9/group. (**D**) Cross-validation of model in **B** acquired through 100 permutation tests; *y*-axis intercepts: *R^2^* = (0.0, 0.21), *Q^2^* = (0.0, –0.30). *n* = 9/group. (**E**) OPLS-DA loadings plot showing the TAG influence on the separation between the HP and C groups. TAGs elevated in HP are displayed on the positive side of the predictive component, while TAGs elevated in C are displayed on the negative. Red circles represent scTAGs. (**F**) Box plots showing the range of saturated scTAGs in C- (blue) and HP-fed (red) individuals). Data are presented as box-whisker plots, with the central box representing the interquartile region and the whiskers the minimum and maximum of the data. Data were analyzed by 2-way repeated-measures ANOVA with post hoc Šidák’s multiple-comparisons test; *P ≤ 0.05, *n* = 9/group.

Nine healthy male subjects completed the trial protocol (Figure 1); baseline characteristics for these volunteers are summarized in Table 1. The subjects consumed an isoenergetic (2 MJ) control (C) meal, high-protein (HP) meal, and a high-fat (HF) meal, and plasma samples were collected hourly over 6 hours. To assess changes in the plasma triglyceride profiles obtained by liquid chromatography–mass spectrometry (LC-MS), data were processed by constructing principal components analysis (PCA) models to identify the distribution of the variables and remove any outliers detected using Hotelling’s T^2^ test. Subsequently, orthogonal projections to latent structures discriminant analysis (OPLS-DA) models were created to discriminate between the 3 groups. In the OPLS-DA models, the time points 3, 4, and 5 hours were utilized, as this provided sufficient time for ingested macronutrients to reach the extrahepatic blood (18). OPLS-DA readily separated the HP group from the C group (*R^2^X* = 0.84, *Q^2^* = 0.47; Figure 2A). However, there was a less clear separation between the HP group and the HF group (*R^2^X* = 0.54, *Q^2^* = 0.32; Figure 2B). Both models were validated using permutation tests, yielding *R^2^X* and *Q^2^* values (*R^2^* = [0.0, 0.56], *Q^2^* = [0.0, –0.63]; *R^2^* = [0.0, 0.21], *Q^2^* = [0.0, –0.30], respectively) lower than the original, hence indicating stable and nonrandom models (Figure 2, C and D). Cross-validation ANOVA (CV-ANOVA) also showed a significant *P* value for both models (*P* = 0.0063 and *P* = 0.0032, respectively).

The loadings plot of the C versus HP model was then used to determine the metabolite features that differ between the groups (Figure 2E). Variable importance in projection (VIP) was utilized to filter important metabolites in the model. The vectors in the projection are regularized such that if all variables were of equal importance, their values would be equal to 1. Therefore, any variable with a VIP value greater than 1 was considered to be a potential discriminant variable. TAGs containing shorter and more saturated FAs (red circles, Figure 2E) were the major VIPs increased in the HP group (Figure 2F).

The TAG profiles were further analyzed by hierarchical clustering, and heatmap representations were obtained from the Spearman’s correlation matrix among metabolites. One of the clusters contained scTAGs with more saturated FAs, indicating that changes in TAG levels were consistent within members of the cluster, with these scTAGs having been previously correlated with DNL and steatosis, as well as cardiovascular disease (19) and risk of developing T2DM (20) (Supplemental Figure 1; supplemental material available online with this article; https://doi.org/10.1172/jci.insight.124819DS1).

### HP feeding in healthy male subjects increases scTAGs in both plasma and the LDL/VLDL fraction.

**Figure 3:**
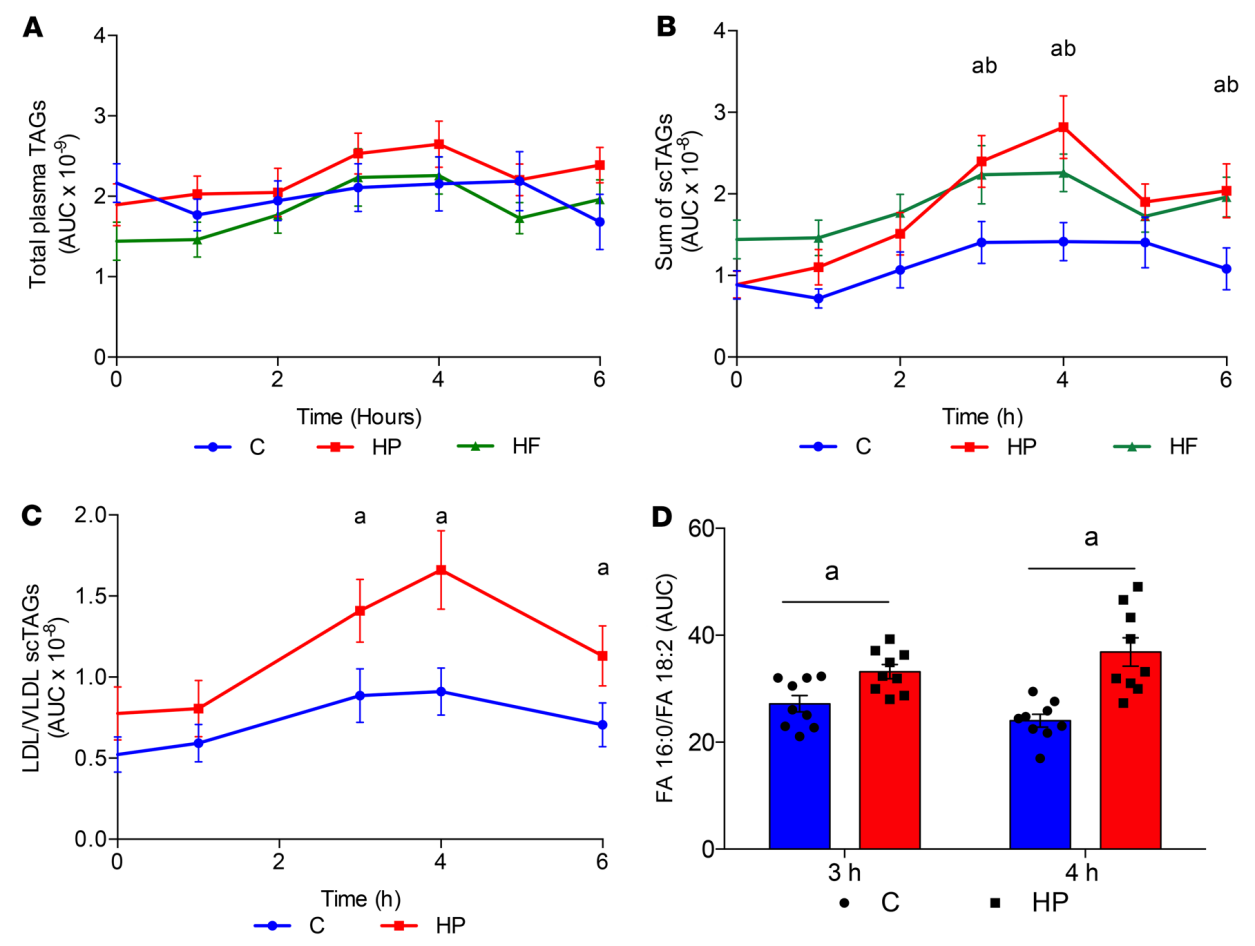
scTAGs are increased in plasma and plasma LDL/VLDL fraction after an HP meal in healthy human subjects. (**A**) Fasting (baseline; 0 hours) and postprandial total TAG abundance in plasma after C (blue), HP (red), and HF (green) meal measured by LC-MS in samples drawn over 6 hours. (**B**) Fasting (baseline; 0 hours) and postprandial sum of scTAG abundance in plasma after C (blue), HP (red), and HF (green) meal measured by LC-MS in samples drawn over 6 hours. (**C**) Fasting (baseline; 0 hours) and postprandial LDL/VLDL scTAG abundance in plasma after C (blue)and HP (red) meal measured by LC-MS in samples drawn over 6 hours. (**D**) DNL index reflected by the ratio of FA 16:0 to FA 18:2 after C (blue) and HP (red) meal at 3 hours and 4 hours. Data are presented as mean ± SEM and analyzed by 2-way repeated-measures ANOVA with post hoc Šidák’s multiple-comparisons test (**A**–**C**) or paired *t* test (**D**); *n* = 9/group. ^a^*P* ≤ 0.05, between C and HP; ^b^*P* ≤ 0.05, between C and HF. No statistically significant difference between HP and HF.

The total amount of triglycerides was not significantly different among the 3 groups (Figure 3A). However, total scTAGs were markedly elevated in HP-fed subjects 3 hours after feeding compared with the same individuals fed the C or HF meal (Figure 3B). After an overnight fast and subsequent feeding with the HP meal, there was an increase in scTAGs up to 4 hours compared with baseline (Figure 3B). There were no significant differences in scTAGs between the baseline and hourly samples in C- and HF-fed subjects (Figure 3B). To determine whether these TAGs were produced by the liver and not the intestine, we extracted LDL/VLDL through a series of precipitation and low-speed centrifugation steps and analyzed their TAG profile. In line with changes in plasma, the sum of scTAGs in the LDL/VLDL fraction was higher in HP-fed than C-fed subjects after 3 hours (Figure 3C). FAs from the meal as well as free FAs (FFAs) are major precursors of lipoprotein TAGs. The C and HP meals did not have any differences in FA composition (Supplemental Table 1) or in plasma FFA content (Supplemental Figure 3), suggesting that these TAGs were synthesized de novo.

The plasma 16:0/18:2n-6 ratio was calculated as an index of DNL, since during DNL plasma TAGs are enriched with 16:0, the primary product of FAS, and depleted in 18:2n-6, an essential FA (21). At 4 hours, where we saw the biggest increase in scTAGs, the 16:0/18:2n-6 ratio was significantly greater after an HP meal than after the C meal (Figure 3D). Increases in DNL during high protein feeding may be explained by increases in insulin. However, the insulin peak at 30 minutes was not significantly different between the C and HP groups (Supplemental Figure 2).

### Glutamate acts as a substrate for the synthesis of TAGs via the TCA cycle and DNL-derived palmitate in AML12 hepatocytes.

**Figure 4:**
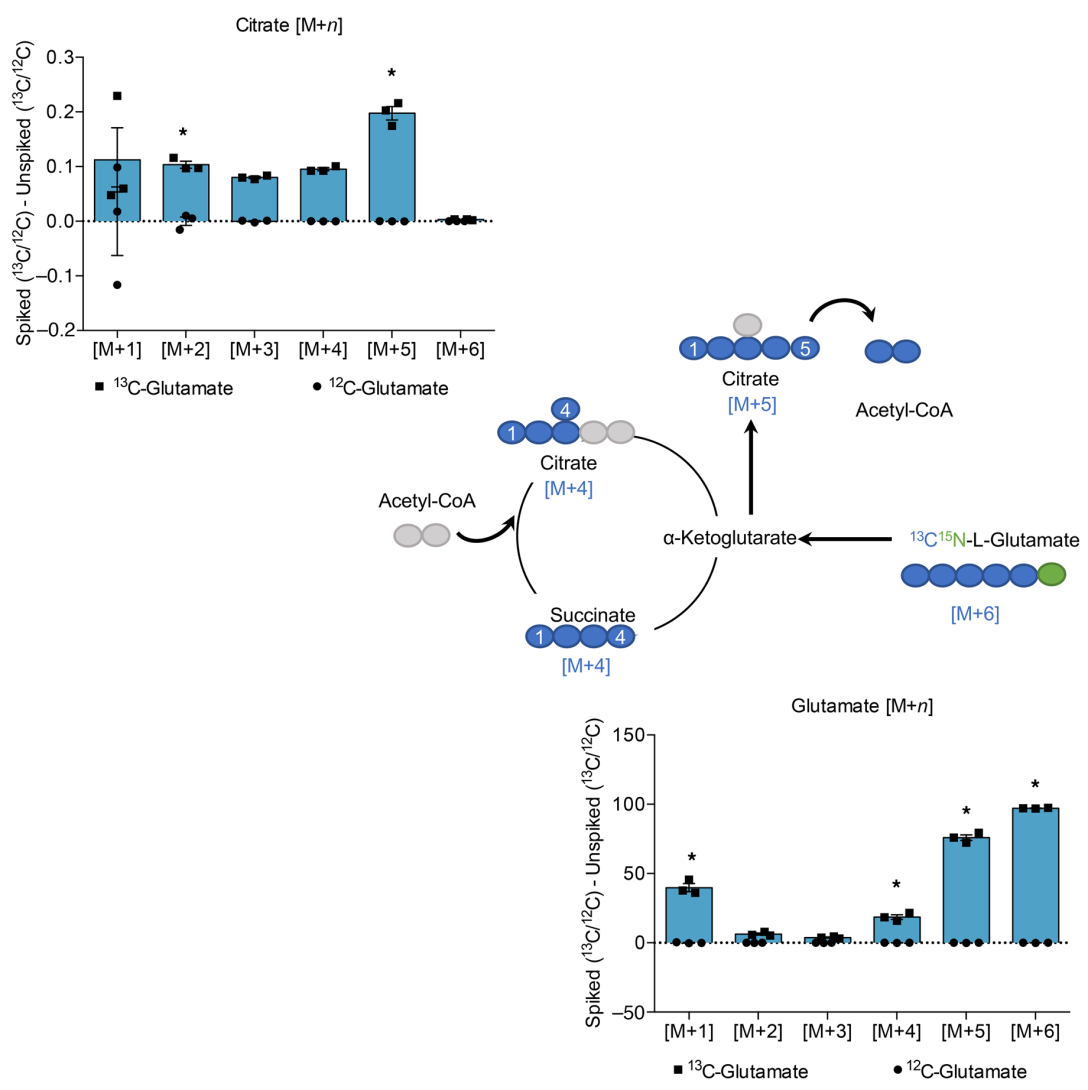
Carbon from ^13^C_5_-labeled glutamate is incorporated into the TCA cycle in AML12 hepatocytes. Labeling in glutamate and citrate, detected by LC-MS over 24 hours after supplementation. Data are presented as mean ± SEM and were analyzed by 2-way ANOVA with post hoc Šidák’s multiple-comparisons test; *P ≤ 0.05, *n* = 3/group.

**Figure 5:**
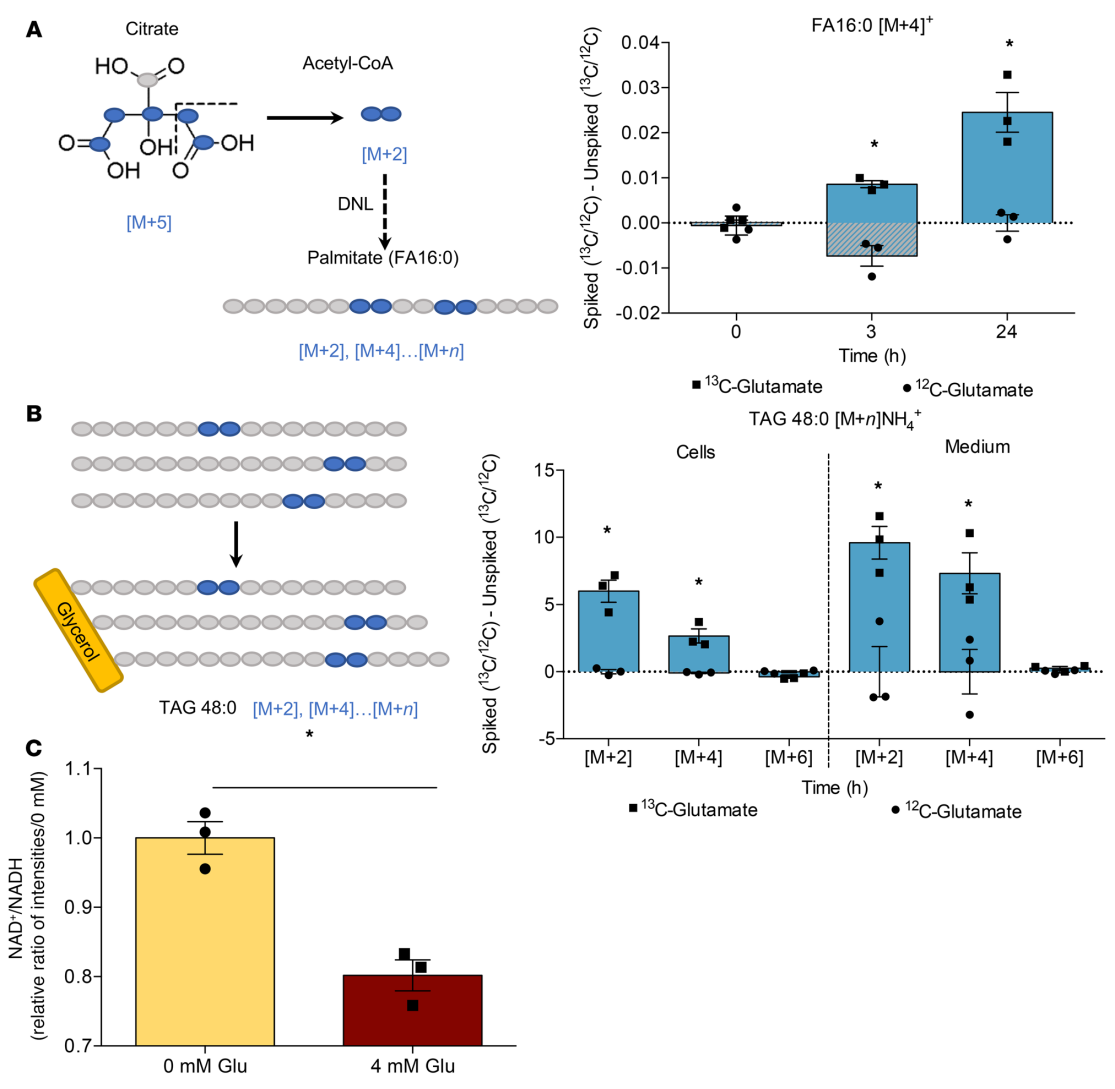
Carbon from ^13^C_5_-labeled glutamate is incorporated into DNL-derived palmitate and triacylglycerols in AML12 hepatocytes. (**A**) Labeling derived from glutamate in the [M+4]^+^ ion of the picolinyl ester of palmitate (FA16:0) generated by DNL, detected by LC-MS over 24 hours after supplementation. (**B**) Labeling derived from glutamate in the [M+2]NH_4_^+^, [M+4]NH_4_^+^, and [M+6]NH_4_^+^ ions of glyceryl tripalmitate (TAG 48:0) produced from DNL-derived palmitate in both cells and medium, detected by LC-MS after 24 hours. (**C**) NAD^+^/NADH at 0 mmol/l and 4 mmol/l glutamate after 24 hours. Data are presented as mean ± SEM and analyzed by 2-way ANOVA with post hoc Šidák’s multiple-comparisons test (**A** and **B**) or unpaired *t* test (**C**); *P ≤ 0.05, *n* = 3/group.

Under physiological conditions, carbohydrates act as the canonical source of substrate for DNL. However, the contribution of amino acids, derived from protein-rich diets, is less well understood beyond the context of oncometabolism. To determine whether amino acids act as substrates for DNL under conditions of typical carbohydrate supply (approximately 5 mmol/l), we supplemented alpha mouse liver 12 (AML12) hepatocytes with ^13^C_5_-labeled glutamate, the most abundant amino acid in the HP meal (Supplemental Table 2), to trace its metabolic fate. The carbon backbone of glutamate may enter the TCA cycle via deamination to α-ketoglutarate. Analysis of subsequent TCA cycle intermediates using mass spectrometry showed that, compared with unlabeled samples, the levels of labeled TCA cycle intermediate citrate [M+2]^+^ and [M+5]^+^ (mass of the unlabeled compound plus 2 or 5 Da for the labeled carbons) were markedly increased in labeled samples over 24 hours (Figure 4). Interestingly, the [M+4]^+^ citrate generated by the addition of unlabeled acetyl-CoA to [M+4]^+^ oxaloacetate cannot supply [M+2]^+^–labeled acetyl-CoA for the synthesis of FAs (22). While citrate is chemically symmetrical, the enzymes citrate synthase (CS) and ACL are regiospecific, such that the acetyl-CoA cleaved by ACL is the same as that added by CS (23, 24). The ^13^C label from glutamate was detected in the [M+4]^+^ ion of palmitate, which was found to be significantly increased in labeled samples 3 hours after supplementation (Figure 5A). Once FAs are synthesized, they are packaged into TAGs and exported from the liver as VLDL. We further detected the label in the scTAG 48:0 both intracellularly and in the cell culture media (Figure 5B).

Thus, we suggest the most likely process by which carbon from glutamate was incorporated into FAs was via reductive carboxylation in the TCA cycle. This reverse flow through the TCA cycle typically occurs during periods of decreased NAD^+^-to-NADH ratio, corresponding to high energy balance, which was detected in glutamate-supplemented samples compared with control (Figure 5C).

Taken together, these data suggest that not only do amino acids provide carbon for use in DNL, the levels of incorporation of amino acid–derived carbon are appreciable even in the presence of physiological glucose concentrations.

### Select amino acids increase scTAGs in AML12 hepatocytes.

**Figure 6:**
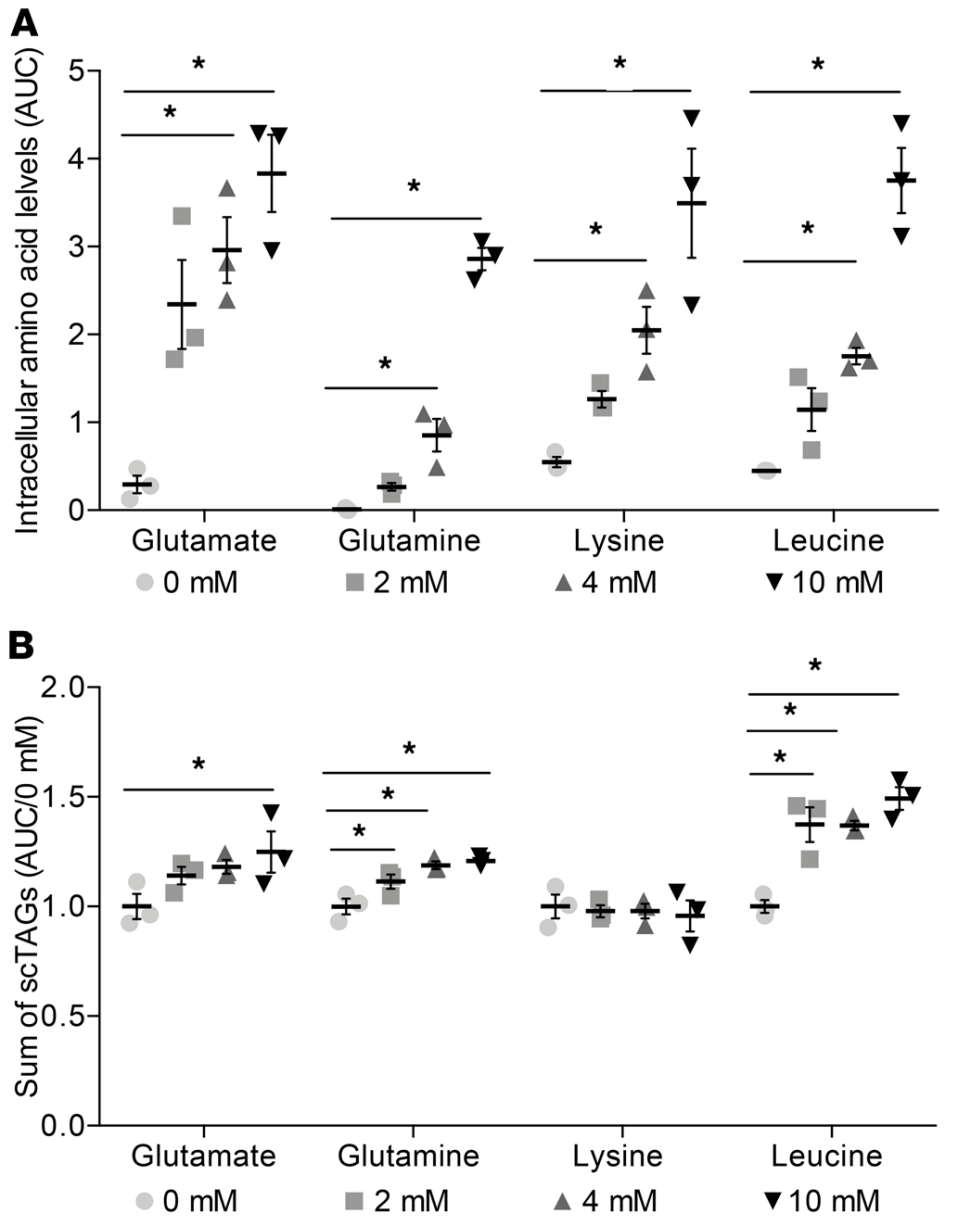
Intracellular scTAGs levels increase dose-dependently in response to glutamate, glutamine, and leucine but not lysine concentrations in AML 12 hepatocytes. (**A**) Amino acid levels in cells in response to specific amino acid treatment at concentrations of 0, 2, 4, and 10 mmol/l measured by LC-MS after 24 hours. (**B**) Sum of scTAG content in cells in response to specific amino acid treatment at concentrations of 0, 2, 4, and 10 mmol/l measured by LC-MS after 24 hours. Data are presented as mean ± SEM and analyzed by 1-way ANOVA with post hoc Dunnett’s multiple-comparisons test; *P ≤ 0.05, *n* = 3/group.

We next sought to determine whether amino acids increase DNL and chose representatives of glucogenic (glutamate and glutamine) and ketogenic (leucine and lysine) amino acids. We supplemented AML12 hepatocytes with increasing concentrations of glutamate, glutamine, leucine, and lysine. As expected, intracellular amino acid levels increased dose dependently in response to the corresponding amino acid increases in media (Figure 6A). Total scTAG levels increased dose dependently in response to glutamate, glutamine, and leucine but not lysine concentrations (Figure 6B), with the greatest effect achieved by supplementation with leucine. Lysine did not increase the levels of scTAGs, and in separate experiments, label from ^13^C_6_-lysine was not detected in palmitate or palmitate-containing triglycerides (Supplemental Figure 4).

### Glutamate increases the expression of DNL genes.

**Figure 7:**
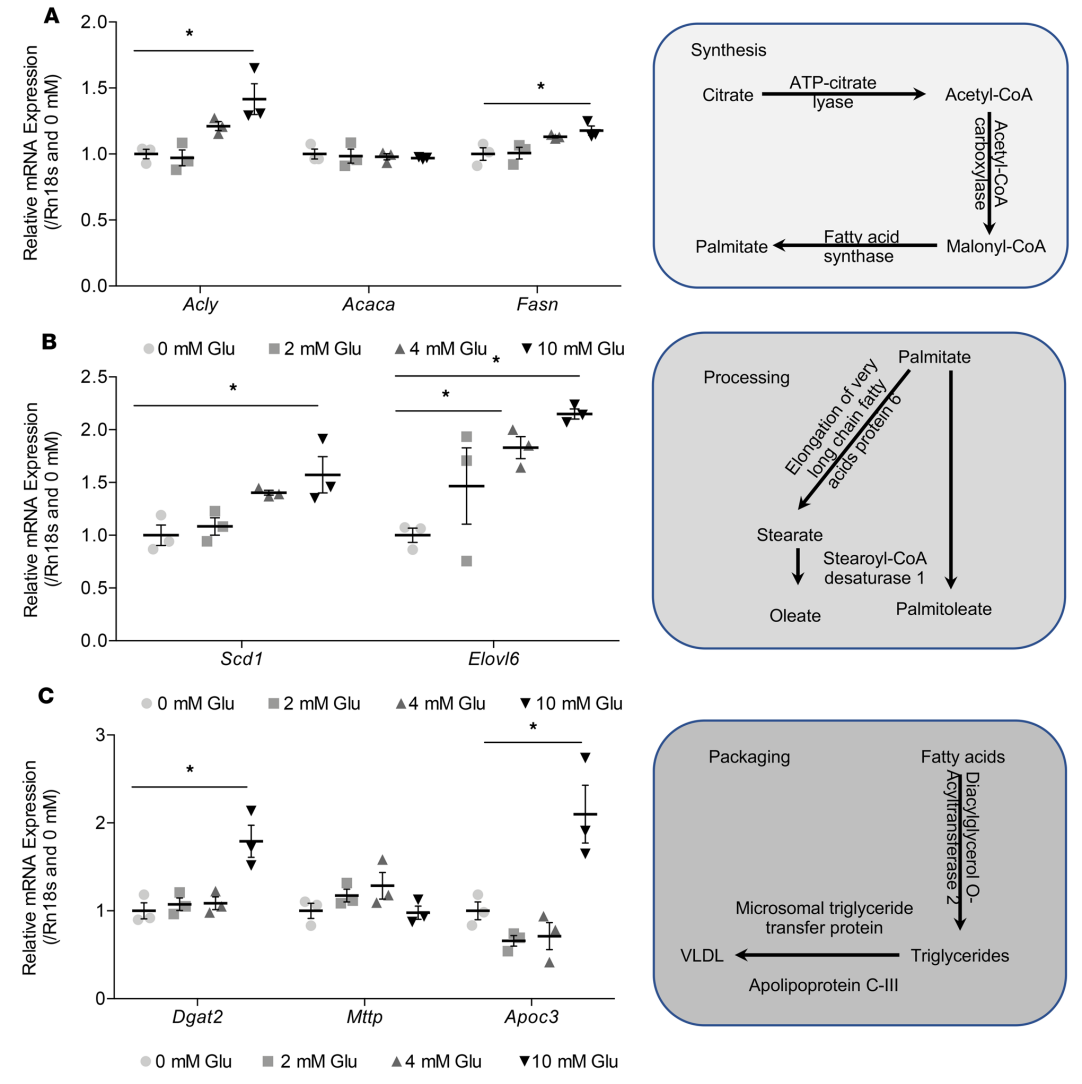
Increasing glutamate concentration increases DNL synthesis, processing, and VLDL packaging gene expression for fatty acids in AML12 hepatocytes. (**A**) qPCR analysis of expression of DNL synthesis genes *Acly*, *Acaca*, and *Fasn* after 24 hours. (**B**) qPCR analysis of expression of processing genes *Scd1* and *Elovl6* after 24 hours. (**C**) qPCR analysis of expression of packaging genes *Dgat2*, *Mttp*, and *Apoc3* after 24 hours. Data are presented as mean ± SEM and analyzed by 1-way ANOVA with post hoc Dunnett’s multiple-comparisons test; *P ≤ 0.05, *n* = 3/group.

The synthesis of VLDL particles may be separated into 3 main processes: synthesis of FAs, elongation and desaturation of FAs, and packing of FAs into TAGs and VLDL. Synthesis of FAs is initiated by ATP-citrate lyase (*Acly*), responsible for lysing citrate to acetyl-CoA. Acetyl-CoA is then carboxylated to malonyl-CoA, via ACC (*Acaca*), which is subsequently utilized by a multifunctional enzyme, FAS (*Fasn*), to synthesize FAs. Increasing levels of glutamate elevated the expression of *Acly* and *Fasn* dose dependently, with a significant increase at 10 mmol/l glutamate after 24 hours (Figure 7A). However, there were no changes in the expression of *Acaca*. Once synthesized, palmitate may be desaturated and/or elongated to palmitoleate and stearate by *Scd1* and *Elovl6*, respectively. Glutamate increased the expression of *Scd1* (10 mmol/l glutamate) and *Elovl6* (4 and 10 mmol/l glutamate; Figure 7B). FAs are then sequentially esterified to glycerol to form triglycerides, with the terminal step catalyzed by diacylglycerol *O*-acyltransferase 2 (*Dgat2*). Microsomal triglyceride transfer protein (*Mttp*) is then responsible for shuttling triglycerides through membranes such that they may associate with apolipoprotein B100 and apolipoprotein C-III (*Apoc3*). Glutamate at 10 mmol/l increased the expression of *Dgat2* and *Apoc3* but not *Mttp* (Figure 7C).

### Profile of expression of DNL genes in response to amino acid supplementation.

**Figure 8:**
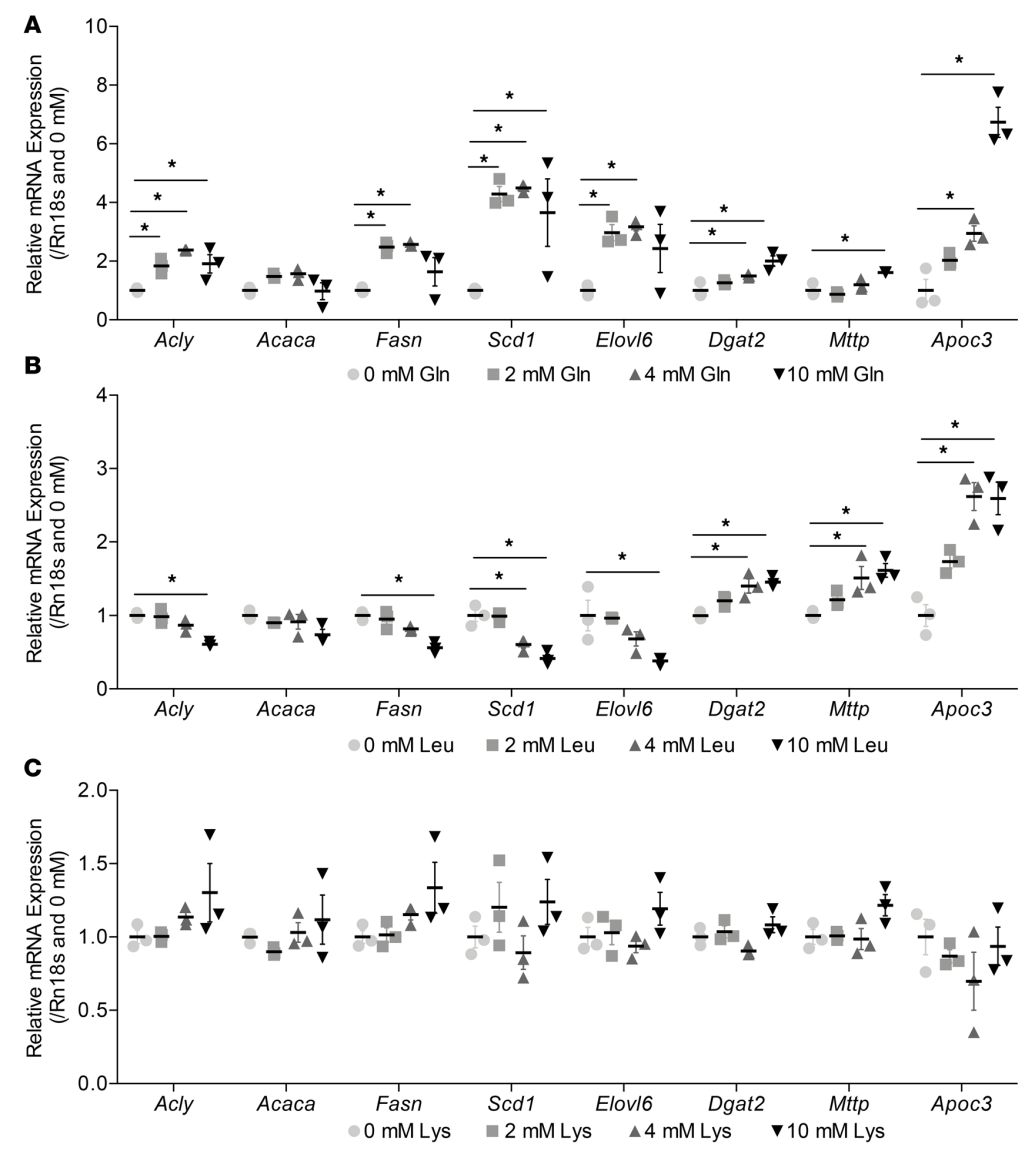
The effect of increasing glutamine, leucine, and lysine concentrations on DNL synthesis, processing, and VLDL packaging gene expression in AML12 hepatocytes. (**A**) qPCR analysis of expression of *Acly*, *Acaca*, *Fasn*, *Scd1*, *Elovl6*, *Dgat2*, *Mttp*, and *Apoc3* in response to 0, 2, 4, and 10 mmol/l glutamine after 24 hours. (**B**) qPCR analysis of expression of *Acly*, *Acaca*, *Fasn*, *Scd1*, *Elovl6*, *Dgat2*, *Mttp*, and *Apoc3* in response to 0, 2, 4, and 10 mmol/l leucine after 24 hours. (**C**) qPCR analysis of expression of *Acly*, *Acaca*, *Fasn*, *Scd1*, *Elovl6*, *Dgat2*, *Mttp*, and *Apoc3* in response to 0, 2, 4, and 10 mmol/l lysine after 24 hours. Data are presented as mean ± SEM and analyzed by 1-way ANOVA with post hoc Dunnett’s multiple-comparisons test; *P ≤ 0.05, *n* = 3/group.

Glutamine at 2 and 4 mmol/l increased expression of the FA synthesis (*Acly* and *Fasn*, but not *Acaca*), processing, and packaging genes (Figure 8A). However, at 10 mmol/l glutamine, expression of *Fasn* and *Elovl6* was no longer significantly increased. Despite the fact that leucine resulted in the greatest increase in scTAGs in hepatocytes, it decreased expression of FA synthesis (except *Acaca*) and processing genes, with significant changes at 10 mmol/l leucine. However, it increased expression of packaging genes dose dependently (Figure 8B). Lysine did not change expression of any genes significantly, in agreement with the finding that lysine did not affect scTAG content in hepatocytes (Figure 8C).

### Glutamate, glutamine, and leucine increase phosphorylation of AKT.

**Figure 9:**
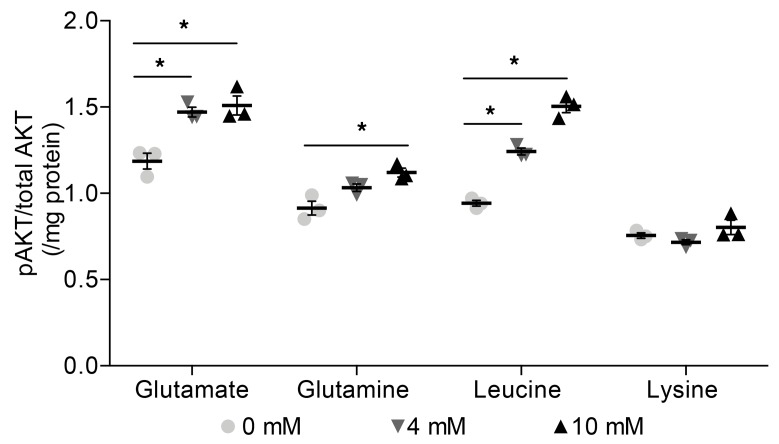
Intracellular pAKT levels increase dose dependently in response to glutamate, glutamine, and leucine but not lysine concentrations in AML12 hepatocytes. Analysis of AKT activation as a function of AKT^pS473^ levels detected by ELISA after 24 hours of amino acid supplementation. Data are presented as mean ± SEM and were analyzed by 1-way ANOVA with post hoc Dunnett’s multiple-comparisons test; *P ≤ 0.05, *n* = 3/group.

Through a partially characterized mechanism, insulin activates the transcription factor SREBP1c to induce the expression of genes required for hepatic DNL. A key node in this signaling cascade is PKB/AKT2, a protein kinase that transduces the signal to downstream effectors, such as mTOR complex 1 (mTORC1). It is known that amino acids activate mTORC1; however, it has only recently been reported that mTORC1 activation in the absence of PKB/AKT2 is not sufficient to stimulate SREBP1c and thus DNL (25). We therefore investigated whether amino acids can act upstream of mTORC1 and activate PKB/AKT2 by quantifying the levels of the active kinase, as a ratio to total PKB/AKT2, via measuring phosphorylation at Ser473. In AML12 hepatocytes, intracellular pPKB/AKT2 levels increased dose dependently in response to glutamate (4 and 10 mmol/l), glutamine (10 mmol/l), and leucine (4 and 10 mmol/l) but not lysine concentrations (Figure 9).

### Glutamine and leucine decrease glucose uptake in AML12 hepatocytes.

**Figure 10:**
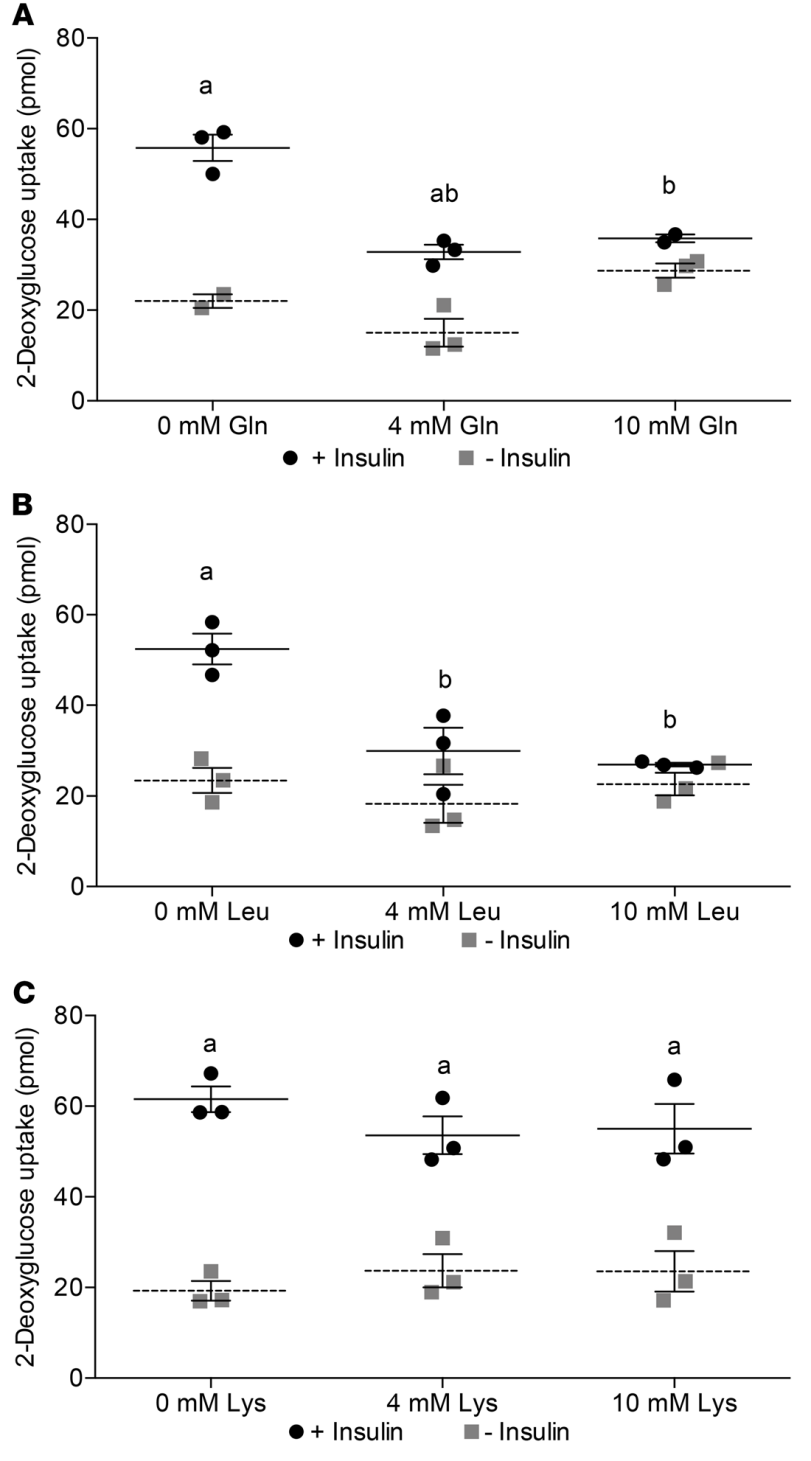
2-Deoxyglucose uptake decreases in response to glutamine and leucine but not lysine in AML12 hepatocytes. (**A**) 2-Deoxyglucose uptake assay on AML12 cells exposed to 0, 4, and 10 mmol/l glutamine for 24 hours. (**B**) 2-Deoxyglucose uptake assay on AML12 cells exposed to 0, 4, and 10 mmol/l leucine for 24 hours. (**C**) 2-deoxyglucose uptake assay on AML12 cells exposed to 0, 4, and 10 mmol/l lysine for 24 hours. Data are presented as mean ± SEM and were analyzed by 2-way ANOVA with post hoc Šidák’s multiple-comparisons test; *n* = 3/group. ^a^*P* ≤ 0.05, between with insulin and without insulin within the same amino acid concentration; ^b^*P* ≤ 0.05, between different amino acid concentrations within insulin treatment versus 0 mmol/l.

As insulin resistance is thought to result, at least in part, from the hyperactivation of mTOR and the subsequent phosphorylation of IRS1 (26), we sought to determine the acute effects of amino acid supplementation on the uptake of the glucose analog 2-deoxyglucose. This molecule is recognized by glucose transporters, phosphorylated within the cell by glucokinase, but cannot be further metabolized and is thus sequestered within the cell. Upon addition of 2-deoxyglucose, insulin-stimulated uptake was significantly lower in both glutamine- and leucine-supplemented cells (Figure 10, A and B, respectively), but not with lysine (Figure 10C).

## Discussion

The latest nutritional transition from traditional to Western-style diets (rich in sugar, fat, and protein from red meat) has led to a growing prevalence of NAFLD and T2DM (3). Hepatic lipid accumulation, in part via upregulated levels of DNL, is mutual to the 2 disorders. While it is well established that high-carbohydrate feeding increases DNL (11), studies of the effect of high-protein feeding in relation to DNL in humans, are limited. Despite the fact that high-protein/low-carbohydrate diets are popular regimes for weight loss in humans to prevent obesity and diabetes, recent studies correlated elevated BCAA levels with insulin resistance and T2DM (12). The precise dietary amino acid composition that may induce DNL is, as yet, unknown.

In the present 3-way crossover study, healthy human males consumed 3 meals (C, HP, and HF) in a randomized order. Only young healthy males were included in this study, as premenopausal females have more variable lipid profiles due to the effects of estrogen; as we were limited in sample size by cost and time constraints, we chose to focus on males for this study. As the volunteers were healthy, the effects observed were a consequence of the parameters of the study, without the confounding factor of disease status or variability in lipid profile. In addition, the meals were designed such that all 3 were isocaloric. Consequently, and unavoidably, the carbohydrate content of the meals varied, which may be considered to confound the results. However, the caloric content was considered the more important determining factor in metabolic processes, including DNL, and we therefore opted to make this parameter a constant 2 MJ.

Lipidomic changes were detected at hours 3, 4, and 5 postprandially, particularly in the TAG class of lipids. Using earlier time points, valid models were not generated, possibly due to the delay between intake of the meal and its subsequent effect on the blood lipidome. Based on the initial multivariate analysis, we opted to focus on a more detailed analysis in the C and HP groups.

We show that dietary amino acids increase scTAGs that have been previously associated with DNL, steatosis (9), and insulin resistance (20). However, it should be noted that these scTAGs are not a direct measure of DNL, and it should be considered a limitation of the study that DNL was not directly measured using stable isotope–based approaches. However, we also show that palmitate may readily be derived from glutamate via DNL, suggesting that amino acids may provide carbon as a substrate for this process under physiological substrate concentrations. We determined differential effects of amino acids on scTAGs as well as the expression of genes required for synthesis, processing, and packaging of FAs, likely involving the action of AKT2 in hepatocytes. We demonstrated that an HP meal, rich in glutamate, increased scTAGs in plasma as well as in the LDL/VLDL fraction in healthy human males. These effects occurred following a 12-hour fast, which typically suppresses DNL (27), and so may underestimate the physiological consequences of an HP meal in terms of DNL. Thus, these results may have general implications for high-protein diets and T2DM. Studies have previously demonstrated that a higher-protein/lower-carbohydrate diet downregulates DNL (28, 29), typically in longer-term dietary interventions. However, the precise amino acid composition of the high-protein diet was not determined, and as we show herein, activation of DNL is specific to certain amino acid treatments. The precise metabolic parameters and time frame of these effects on DNL remain to be fully elucidated.

A master regulator of cell growth, mTORC1, is responsible for sensing nutrient signals, particularly amino acids (30). The mTORC1 signaling pathway integrates insulin signals, through its phosphorylation by PI3K/AKT2 (31). Activation of mTORC1 leads to the nuclear localization of liver X receptor α (LXRα), whereupon it heterodimerizes with the retinoid X receptor (RXR) to induce the expression of lipogenic genes as well as SREBP1c (32). mTORC1 also mediates the nuclear translocation of SREBP1c, whereby it induces the transcription of genes required for DNL (33). However, it has recently been reported that mTORC1 activation in the absence of AKT2 is not sufficient to stimulate SREBP1c, and thus DNL (25), suggesting that amino acids regulate an upstream node in the pathway, or act in an as-yet-undetermined manner. Our data show that select amino acids — glutamate, glutamine, and leucine but not lysine — activated AKT2. This may also indicate that specific amino acids may regulate mTORC2, responsible for the activation of AKT2 and subsequent induction in expression of genes required for DNL, in support of a signaling pathway proposed by Tato et al. (34). Amino acids, branched-chain in particular, have been linked with insulin resistance. The molecular basis is the activation p70 S6k by amino acids (35), a downstream kinase of mTORC1. This in turn phosphorylates the insulin receptor substrate at multiple serine/threonine residues, thereby attenuating glucose uptake. Persistent activation of p70 S6k by excessive amino acid concentrations may therefore lead to insulin resistance (26). Herein, we demonstrate that glutamine and leucine decrease glucose uptake, while lysine does not.

In support of our finding that specific amino acids activate PKB/AKT2, we also demonstrate that the same amino acids increased scTAG content in hepatocytes. Leucine resulted in the greatest increase in scTAG content, followed by glutamine and, last, glutamate. Both glutamate and glutamine increased expression of genes required for the synthesis, processing, and packaging of FAs. Perhaps surprisingly, leucine and the highest concentration of glutamine decreased expression of genes required for synthesis and processing of FAs but increased expression of genes required for packaging. This suggests a possible negative feedback mechanism, whereby accumulation of high levels of scTAGs or other intermediate species (such as fatty acyl-CoAs) in the liver inhibit expression of these genes, but not expression of genes required for subsequent processing and packaging. Indeed, studies demonstrate that unsaturated FAs lower levels of mRNA for SREBP1c by accelerating its degradation (36), while longer-chain polyunsaturated FAs are potent inhibitors of ACC (37). Our findings also suggest that FA synthesis and processing genes are differentially regulated to packaging genes. SREBP1c induces expression of genes required for the synthesis (*Acly*, *Acaca*, and *Fasn*) and processing (*Scd1* and *Elovl6*) of FAs (38). On the other hand, packaging genes (*Mttp* and *Apoc3*) have been shown to be regulated by the FoxO1 transcription factor (39). FoxO1 is responsible for expression of the gene encoding functional MTP, a protein responsible for the packaging of lipids with nascent apolipoprotein B (only present in VLDL) in the endoplasmic reticulum. The action of MTP on the growing VLDL particle is a rate-limiting step in their formation and release into the blood. Elevated levels of MTP increase the secretion of VLDL by the liver and correlate with the pathophysiology of insulin resistance and T2DM (40). FoxO1 is itself negatively regulated by insulin, which causes its phosphorylation and nuclear exclusion (41, 42). In cases of impaired insulin regulation, this may result in uncontrollable levels of MTP (40) expression. The production of hepatic, but not intestinal, apolipoprotein CIII is also controlled by FoxO1 via the –498/–403 element in the *apoC3* promoter (41, 43). Two separate insulin signaling axes, PI3K/PKB/AKT2 and MAPK(erk), may be responsible for this inhibition. While there is evidence supporting a role for both axes (44, 45), our data suggest that PI3K/PKB/AKT2 is not involved in this inhibition, as the expression of *Mttp* and *apoC3* is increased in the presence of increased PKB/AKT2. This is consistent with the work of Au et al., which demonstrated that insulin inhibits the transcription of *Mttp* via the MAPK(erk) signaling cascade, but not that of PI3K (46). In the context of insulin resistance and the development of fatty liver via dysregulated rates of DNL, it might be considered hepatoprotective to increase the rate at which liver-derived lipoproteins are exported, at least in the shorter term. However, it is unlikely that prolonged hypertriglyceridemia, brought about by chronic insulin resistance, has equally beneficial effects on long-term metabolic health.

In summary, our data indicate that high-protein feeding increases scTAG content in post-fasting, healthy human male plasma, indicative of hepatic DNL, and in liver-derived lipoproteins. We also demonstrate that specific amino acids are responsible for these effects in a cell culture system. The human study utilized only 9 male subjects; this should be expanded in a larger and more diverse cohort before generalized applicability is possible. As dietary trends have shifted toward high-carbohydrate/high-fat consumption, and high-protein diets are popularized as healthier alternatives, the data herein suggest that this is a more complex consideration. Therefore, advocating the consumption of protein in the treatment of diabetes and obesity requires a more profound understanding of the roles of amino acids in the context of hepatic DNL in larger cohort studies.

## Methods

### Human intervention protocol

#### General design.

The study was divided into recruitment, prescreening questionnaire, and screening visit and 3 separate study days. Nine healthy, nonsmoking men took part in a randomized, 3-way crossover study. Twenty-four hours before each trial day, the diet and activity of each participant were monitored. On the trial day, participants attended the Medical Research Council — Elsie Widdowson Laboratory (MRC-EWL) for 7 hours and received one of the 3 isoenergetic mixed meals in a randomized order. Nine subjects were randomized into one block. Randomization was generated using a computerized program that generates random permutations. Volunteers were randomized at a 1:1:1 ratio, such that each volunteer consumed all 3 meals within a period of 6 weeks. The isoenergetic meals comprised: control (C; 15% protein, 40% fat, 45% carbohydrate), high-protein (HP; 32% protein, 33% fat, 35% carbohydrate), and high-fat (HF; 14% protein, 62% fat, 24% carbohydrate).

#### Screening visit.

Screening measurements and a blood sample were taken during the screening visit. Participants were also requested to complete a 24-hour food diary. After an overnight 12-hour fast, anthropometric measurements (weight, height, and blood pressure) and a fasting blood sample were taken. The fasting blood sample was taken by venipuncture for analysis of full blood count, liver function, glucose, and insulin and lipid profile, including total cholesterol, HDL, LDL, and triglycerides. These measurements were used to determine whether participants met the inclusion criteria of the study (Table 2).

#### Study day protocol.

Participants arrived in the morning after a 12-hour overnight fast. Participants were cannulated via the antecubital vein of one arm and blood samples were collected, 10 minutes apart. Participants then consumed, within 15 minutes of onset of eating, one of the isoenergetic meals. Subsequently blood samples were collected over a 6-hour period. During the 6-hour period, food consumption was not allowed, and only water was permitted ad libitum after 3 hours had elapsed. Blood samples were collected at 12 time points at various intervals throughout the 6-hour period (T1–T6) to perform mass spectrometry and determine blood lipid changes after an HP meal.

### LDL/VLDL purification

Purified LDL/VLDL fractions were obtained using an LDL/VLDL Purification kit (Ultracentrifugation Free; Cell Biolabs Inc.). To 200 μl plasma on ice, 10 μl dextran solution and 100 μl precipitation solution A were added. The samples were incubated for 5 minutes on ice before centrifuging (6000 *g*) for 10 minutes at 4°C. The remaining pellet (containing LDL/VLDL) was resuspended in 80 μl bicarbonate solution and centrifuged (6000 *g*) for 10 minutes at 4°C. The supernatant was transferred to a new tube and mixed thoroughly with 1 ml of 1× precipitation solution B and centrifuged (6000 *g*) for 10 minutes at 4°C. The pellet was resuspended in 40 μl of 5% sodium chloride solution, mixed thoroughly with 1 ml of 1× precipitation solution C, and centrifuged (6000 *g*) for 10 minutes at 4°C. The above step was repeated and the pellet resuspended in 100 μl sodium chloride solution. To the mixture was added 16 μl of dextran removal solution before incubation for 1 hour at 4°C and then centrifuging (6000 *g*) for 10 minutes at 4°C. The supernatant (containing purified LDL/VLDL) was recovered and stored at –80°C. After the purified mixture of LDL/VLDL fractions was acquired, metabolites were extracted from these fractions as described below and analyzed by LC-MS of the lipid fraction.

### Metabolite extractions

Metabolites were extracted from blood plasma/cells using the modified method of Folch and colleagues (47). Briefly, 15 μl blood plasma or pelleted cells was mixed with chloroform/methanol (2:1, 750 μl), including a mixture of internal standards labeled with deuterium (TAGs 45:0-d29, 48:0-d31, and 54:0-d35; Qmx Laboratories Ltd.). Samples were sonicated for 15 minutes, and water was added (300 μl). Samples were then centrifuged at 13,000 *g* for 20 minutes. The organic (upper layer) and aqueous phases (lower layer) were separated. The organic samples, containing the lipid extracts, were dried under a stream of nitrogen gas, while the aqueous samples were dried in a CentriVap Centrifugal Concentrator with attached cold trap (78100 series, Labconco).

### LC-MS of the organic fractions

The organic fraction was reconstituted in 100 μl chloroform/methanol (1:1) and 10 μl added to 90 μl IPA/acetonitrile/water (2:1:1). Analysis of the fractions was performed using an LTQ Orbitrap Elite Mass Spectrometer (Thermo Scientific). In positive mode, 5 μl sample was injected onto a C18 CSH column, 1.7-μm pore size, 2.1 mm × 50 mm (catalog 186005296, Waters) which was held at 55°C in a Dionex Ultimate 3000 ultra-high performance liquid chromatography system (UHPLC; Thermo Fisher Scientific). A gradient (flow rate, 0.5 ml/min) of mobile phase A (acetonitrile/water 60:40, 10 mmol/l ammonium formate) and B (LC-MS–grade acetonitrile/isopropanol (IPA) 10:90, 10 mmol/l ammonium formate) was used. In negative ion mode, 10 μl of the sample was injected and 10 mmol/l ammonium acetate was used as the additive to aid ionization. In both positive and negative ion mode, the gradient began at 40% B; increased to 43% B at 0.8 minutes, 50% B at 0.9 minutes, 54% B at 4.8 minutes, 70% B at 4.9 minutes, and 81% B at 5.8 minutes; spiked at 99% B at 8 minutes for 0.5 minutes; and subsequently returned to the starting conditions for another 1.5 minutes to reequilibrate the column. The HPLC was coupled to an electrospray ionization (ESI) source before entering the mass spectrometer. The data were collected in both positive and negative ion mode with a mass range of 110–2000 *m*/*z*. Default instrument-generated optimization parameters were used. Tandem MS was performed, using normalized collision energy, to fragment the intact lipids listed in Supplemental Table 3 in order to identify the fatty acyl chains contained within.

### Picolinyl ester FA derivatization of organic fractions for LC-MS

Picolinyl esters of FAs (PEFAs) were produced using a modification to a method published previously (48) to measure total FA content. To each dried organic extract 200 μl of 10 μmol/l deuterated internal standard mix (containing FAs 13:0, 15:0, 17:0, and 20:0) were added and dried under nitrogen. Each sample was fully resuspended in 200 μl oxalyl chloride (2 mol/l in dichloromethane, catalog 310670, Sigma-Aldrich) and incubated at 65°C in a heating block for 10 minutes, achieving cleavage of FAs from complex lipids and activating the carboxylic group. Samples were then dried under nitrogen, resuspended in 200 μl dichloromethane (catalog 270997, Sigma-Aldrich), and dried again. Each dried residue was then resuspended in 150 μl of 1% 3-hydroxymethylpyridine (catalog P66807, Sigma-Aldrich) in acetonitrile and incubated at room temperature for 5 minutes to produce derivatized FAs. The dichloromethane resuspension was repeated to ensure unreacted 3-hydroxymethylpyridine had evaporated, dried under nitrogen, and stored at –80°C until analysis.

### Analysis of total FAs by triple quadrupole mass spectrometry

PEFAs were reconstituted in 100 μl 2:1 methanol/water and sonicated for 15 minutes. Samples were then centrifuged for 15 minutes at 13,000 *g* to pellet any remaining debris. A 2-μl injection volume of the resulting solution was analyzed on a TSQ Quantiva Triple Quadrupole mass spectrometer attached to a Vanquish UHPLC system. Chromatographic separation was achieved on an Acquity UPLC BEH C18 1.7 μm × 2.1 mm × 50 mm column (catalog 186002350, Waters). Mobile phase A was 100% water with 0.1% formic acid, and mobile phase B was 50:50 acetonitrile/IPA with 0.1% formic acid. The chromatography gradient started at 30% B for 2.33 minutes, increased to 100% B over 1.34 minutes, and decreased to 30% B for 1.23 minutes (to reequilibrate the column) at a flow rate of 0.735 ml/min. Default instrument-generated optimization parameters were used. Xcalibur Software (Thermo Scientific) was used to identify peaks, process mass spectra, and normalize data to the closest-eluting internal standard.

### Data processing for open-profiling lipidomics

Samples obtained from human subjects were acquired in 2 analytical batches with the analytical method described above, along with a set of quality controls (QCs, obtained by pooling 15 μl of all samples). The resulting raw data files were converted into mzML format using the tool MSConvert of Proteowizard software (49, 50), and further processed within the R environment (51) with the libraries IPO, XCMS, and CAMERA (52–54) to perform parameter optimization based on the QC samples, peak extraction, grouping, retention time correction, and annotation of adducts and isotopes. The output from these preprocessing steps was exported as a CSV file and imported into an adapted implementation of the KniMet (55) pipeline for postprocessing. The LOESS batch correction utility was used to normalize for differences among the 2 analytical batches (based on QCs for intra-batch correction, and on all samples for inter-batch). Features were filtered first based on their presence in the QC samples on the 2 separate batches, with the QC-based feature filtering functionality (thresholds for missing values and relative standard deviation [RSD]/coefficient of variation [CV] = 50% and 20%, respectively), while median peak area comparison, as previously described by Dunn and coworkers (56), was used on the merged dataset. A summary of the CVs for key lipids is presented in Supplemental Table 3. Metabolites were annotated based on accurate mass using a library built from the LIPID MAPS mass spectrometry combinatorial expansion package (57). Finally, the data matrix to be utilized for multivariate statistical analysis was subjected to missing values imputation with the KniMet MVI-KNN tool.

### Growth of AML12 hepatocytes

AML12 cells were purchased from ATCC (CRL-2254) and cultured in 1:1 DMEM and Ham’s F12 medium (Thermo Fisher Scientific), supplemented with 10% FBS, 1% penicillin/streptomycin (100 U/ml and 100 μg/ml, respectively), 1% insulin-transferrin-selenium (ITS; 10 mg/l, 5.5 mg/l, and 6.7 μg/l, respectively), and dexamethasone (100 μmol/l) at 37°C in 5% CO_2_. Cells were removed from liquid nitrogen, thawed rapidly, and initially cultured in a T25 flask (catalog 690175, Greiner). Medium was changed every 2 days, and upon reaching confluence, cells were subcultured in T75 flasks (catalog 7340290, VWR) at a 1:3 ratio. Cells were plated at a density of 50,000 cells/well in collagen 1–coated 12-well plates (catalog 7340295, VWR) and grown to confluence in maintenance medium. Cells were then supplemented with low glucose (7.60 mmol/l) with unlabeled l-glutamate (4 mmol/l) and dialyzed FBS (Thermo Fisher Scientific) for 2 days. After switching medium, cells were serum starved for 20 hours in low-glucose medium supplemented with unlabeled l-glutamate (4 mmol/l).

### AML 12 cell ^13^C_5_-^15^N-l-glutamate labeling procedure

Cells were supplemented with glucose (7.60 mmol/l, *n* = 3) with unlabeled-l-glutamate (4 mmol/l) or glucose (7.60 mmol/l, *n* = 3) with ^13^C_5_^15^N-l-glutamate (4 mmol/l, *n* = 3). Based on previous experiments, the cells were allowed 3 hours so that the ^13^C_5_-^15^N-l-glutamate could reach an isotopic steady state in the TCA cycle. Cells and media were harvested at 0, 3, and 24 hours.

### Dose response of glutamate, glutamine, leucine and lysine in AML12 hepatocytes

Cells were supplemented with low glucose (7.60 mmol/l, *n* = 3) without the specific amino acid (0 mmol/l) or with increasing levels of amino acid (2, 4, and 10 mmol/l, *n* = 3/amino acid). Cells and media were harvested after 24 hours.

### Harvesting of AML12 cells for metabolomics

For cells and media undergoing metabolomic analyses, 900 μl medium was taken from each well and frozen at –80°C. Each well was then washed with 1 ml of 0.9% saline, and cells were lifted from the plates by adding 0.5 ml trypsin (10× trypsin-EDTA, catalog 25300045, Invitrogen). Each well was rewashed with 0.5 ml of 0.9% sterile-filtered saline, and the resulting solution was transferred into a 2-ml microcentrifuge tube and centrifuged at 13,000 *g* for 10 minutes to pellet the cells. The supernatant was removed, and the pellet was resuspended in 750 μl of a 2:1 chloroform/methanol solution to prevent enzymatic degradation of metabolites and frozen at –80°C.

### Analysis of aqueous metabolites by triple quadrupole mass spectrometry

Metabolites were extracted as described above, and aqueous extracts were reconstituted in 50 μl of 10 mmol/l ammonium acetate in water before TCA cycle intermediates were separated using reversed-phase liquid chromatography using a Vanquish UHPLC attached to a TSQ Quantiva triple quadrupole mass spectrometer. Multiple reaction monitoring was used in conjunction with positive/negative ion mode switching utilizing the optimized mass transitions. A C18-PFP column (150 mm × 2.1 mm, 2.0 μm; ACE) was utilized at a flow rate of 0.5 ml/min, with a 3.5-μl injection volume. For chromatography on the UHPLC system, mobile phase A was 0.1% formic acid in water, and mobile phase B was 0.1% formic acid in acetonitrile. The gradient started at 30% B, increased to 90% B at 4.5 minutes for 0.5 minutes, and returned to the starting conditions for a further 1.5 minutes to reequilibrate the column. Mass transitions of each species were as follows (precursor > product): d_5_-l-proline 121.2 > 74.2; d_8_-l-valine 126.1 > 80.2; d_10_-l-leucine 142.0 > 96.2; l-glutamate [M] 148.0 > 84.2; l-glutamate [M+1] 149.0 > 85.2; l-glutamate [M+6] 154.1 > 89.1; citrate 191.0 > 111.0; citrate [M+1] 192.0 > 112.0; citrate [M+2] 193.0 > 113.0; citrate [M+3] 194.0 > 114.0; citrate [M+4] 195.0 > 114.0; citrate [M+5] 196.0 > 115.0; citrate [M+6] 197.0 > 116.0. Collision energies and radio frequency (RF) lens voltages were generated for each species using the TSQ Quantiva optimization function. Xcalibur Software was used to identify peaks, process mass spectra, and normalize data to the closest-eluting internal standard.

### NAD^+^/NADH assay

Briefly, cells were lysed using NAD^+^/NADH extraction buffer, and enzymes that consumed NADH were removed by filtration through a 10 kDa spin column (Abcam, ab93349). NAD^+^/NADH was measured according to the manufacturer’s protocol (Abcam, ab65348).

### RNA extraction and purification from AML12 hepatocytes

Total RNA was extracted and purified from hepatocytes using an RNeasy Mini Kit (QIAGEN) according to the manufacturer’s specifications. Purified RNA concentration was quantified at 260 nm using a NanoDrop 100 (Thermo Fisher Scientific).

### cDNA production by reverse transcription

Each purified RNA sample was diluted with RNase-free water to a final concentration of 100 ng/μl. cDNA synthesis and genomic DNA elimination in RNA samples were performed using an RT^2^ First Strand Synthesis kit (QIAGEN) according to the manufacturer’s specifications. The reactions were stored at –20°C prior to real-time PCR analysis.

### Quantitative-PCR

The relative abundance of transcripts of interest was measured by quantitative-PCR (qPCR) in RT^2^ SYBR Green Mastermix (QIAGEN) with a StepOnePlus detection system (Applied Biosystems). The SYBR Green qPCR Mastermix contained HotStart DNA Taq Polymerase, PCR Buffer, dNTP mix (dATP, dCTP, dGTP, dTTP), and SYBR Green dye. Before adding cDNA to each well of the 96-well plate, cDNA was diluted in RNase-free water to final concentration 8 ng/μl. PCR component mix was prepared by mixing 10 μl SYBR Green qPCR Mastermix with 0.6 μl of 10 μmol/l target primers (forward and reverse; 6 pmol/reaction) and 4.4 μl RNase-free water. To each well of a 96-well plate, 5 μl cDNA (total amount 40 ng) and 15 μl PCR components mix were added. The plate was centrifuged at 1000 *g* for 30 seconds to ensure that the contents were mixed and to remove any bubbles present in the wells. The plate was placed in the real-time cycler with the following cycling conditions: 10 minutes at 95°C for 1 cycle to activate HotStart DNA Taq Polymerase; 15 seconds at 95°C and 1 minute at 60°C to perform elongation and cooling for 40 cycles. RT^2^ qPCR Primer Assays for mouse *Rn18s*, *Fasn*, *Acaca*, *Acly*, *Elvol6*, *Scd1*, *Dgat2*, *Mttp*, and *Apoc3* were purchased from QIAGEN. Expression levels were normalized to the endogenous control, *Rn18s*, using the ΔΔC_t_ method, and fold changes reported were relative to the control group in the dose response (no extra supplemented amino acid in the media).

### Preparation of cell lysates

Cell pellets were lysed in 100 μl cell extraction buffer (10 mmol/l Tris, 100 mmol/l NaCl, 1 mmol/l EDTA, 1 mmol/l EGTA, 10 mmol/l NaF, 20 mmol/l Na_4_P_2_O_7_, 20 mmol/l Na_3_VO_4_, 1% Triton X-100, 10% glycerol, 0.1% sodium dodecyl sulfate, 0.5% deoxycholate, 1 mmol/l phenylmethylsulfonyl fluoride, complete protease inhibitor tablet, and 1% of each phosphatase cocktail inhibitor 2 and 3) for 30 minutes, vortexing at 10-minute intervals. The lysate was centrifuged at 13,000 *g* for 10 minutes at 4°C, and the supernatant was collected and stored at –80°C.

### Cell AKT^pS473^ and AKT (total) quantification by ELISA

PKB/AKT concentrations were measured using commercial assay kits (Invitrogen). The protocol was followed according to the manufacturer’s specifications. The background absorbance was subtracted from all data points, including standards, and a standard curve was generated. The unknown concentrations were read from the standard curve, and the concentrations were multiplied by the appropriate dilution factor. Values were normalized to protein concentration using a reducing agent–compatible bicinchoninic acid protein assay, and values of AKT^pS473^ were normalized to AKT (total).

### 2-Deoxyglucose uptake assay

2-Deoxyglucose uptake was measured using a commercial assay kit (Abcam, ab136955). The protocol was followed according to the manufacturer’s instructions.

### Statistics

To calculate the sample size of the study, a paired *t* test between any 2 comparisons with adjusted multiple comparisons was used. Based on 80% study power, and an adjusted α value of 0.017, 9 subjects were needed for this study.

The distribution of the lipid species did not deviate significantly from a normal distribution (D’Agostino-Pearson omnibus normality test). Multivariate statistical analyses were performed in SIMCA-P software, version 13.0 (Umetrics). All variables were UV scaled and subjected to principal component analysis (PCA) coupled with Hotelling’s T2 test to evaluate the distribution of the observations and identify any possible outliers. Subsequently, samples were classified based on the diet, and a supervised OPLS-DA model was developed to maximize separation between the different classes. The models were validated by a permutation test (*n* = 100), and their significance (*P* ≤ 0.05) was assessed by submitting the scores of the models to a CV-ANOVA test. Loadings plots and the VIP were acquired for each model to determine which variables drive the separation between classes (threshold limit >1.0). Once metabolites were annotated in KniMet and analyzed in SIMCA, the most discriminant variables were TAGs. Fragmentation (as described above) was performed on a group of TAGs to confirm their identity (Supplemental Table 4).

These TAGs were subsequently visualized using univariate statistics. The extent to which the model fits and predicts the data is represented by *R^2^X* and *Q^2^X*, respectively. For univariate statistical analyses, data were visualized using GraphPad (Prism 5.2; GraphPad Software). All data are expressed as mean with SEM. In GraphPad, 1- or 2-way ANOVA was performed where appropriate to determine significant differences between experimental groups. For 1-way ANOVA, Dunnett’s post hoc multiple-comparisons test was performed, while for 2-way ANOVA, Šidák’s post hoc multiple-comparisons test was used. Differences between experimental groups were considered to be statistically significant at *P* ≤ 0.05.

Spearman’s correlation among the metabolic features annotated as TAGs deriving from open-profiling lipidomics was calculated and visualized within R (51) using the cor function of the stat package and the heatmap.2 function of the gplots library (58), respectively.

### Study approval

The protocol of the present human study was approved by both the internal research review board, MRC – EWL, and the Cambridge South Local Research Ethics Committee, Cambridge, United Kingdom. Written informed consent was received from the participants prior to their inclusion in the study.

## Author contributions

EC and XL conducted the human study. EC, XL, MH, and EO collected human samples. EC and SL performed data processing and analysis. EC and TA devised and conducted cell culture experiments. EC, TA, BDM, and JAW performed LC-MS. XL and JLG designed the human study. EC, TA, and JLG interpreted the data and wrote the manuscript.

## Supplementary Material

Supplemental data

ICMJE disclosure forms

Supplemental Data Set 1

## Figures and Tables

**Table 2:**
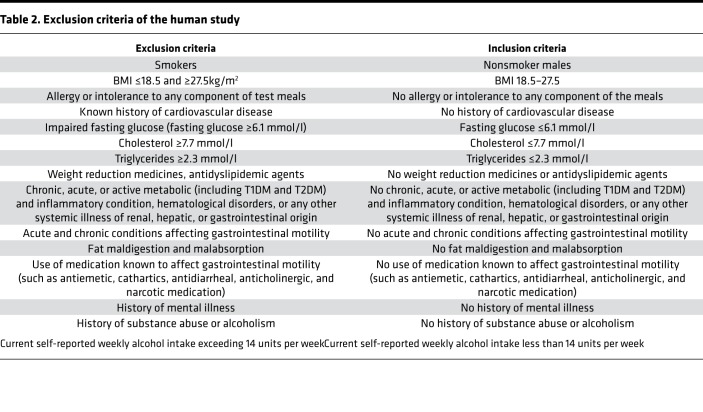
Exclusion criteria of the human study

**Table 1:**
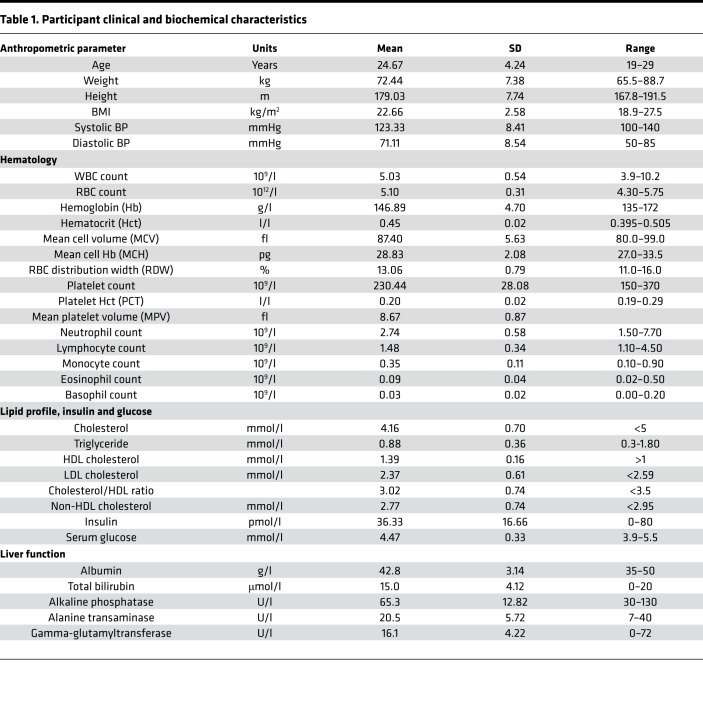
Participant clinical and biochemical characteristics

## Version 1 05/30/2019

In-Press Preview

## Version 2 06/20/2019

Electronic publication
