# Supplementary material for: A randomized 3-way crossover study indicates that high-protein feeding induces de novo lipogenesis in healthy humans
Source: JCI Insight. 2019 May 30;4(12):e124819. doi: 10.1172/jci.insight.124819 (PMC6629161; doi:10.1172/jci.insight.124819)
Supplement: Supplemental data [file jciinsight-4-124819-s050.pdf]

Supplemental material

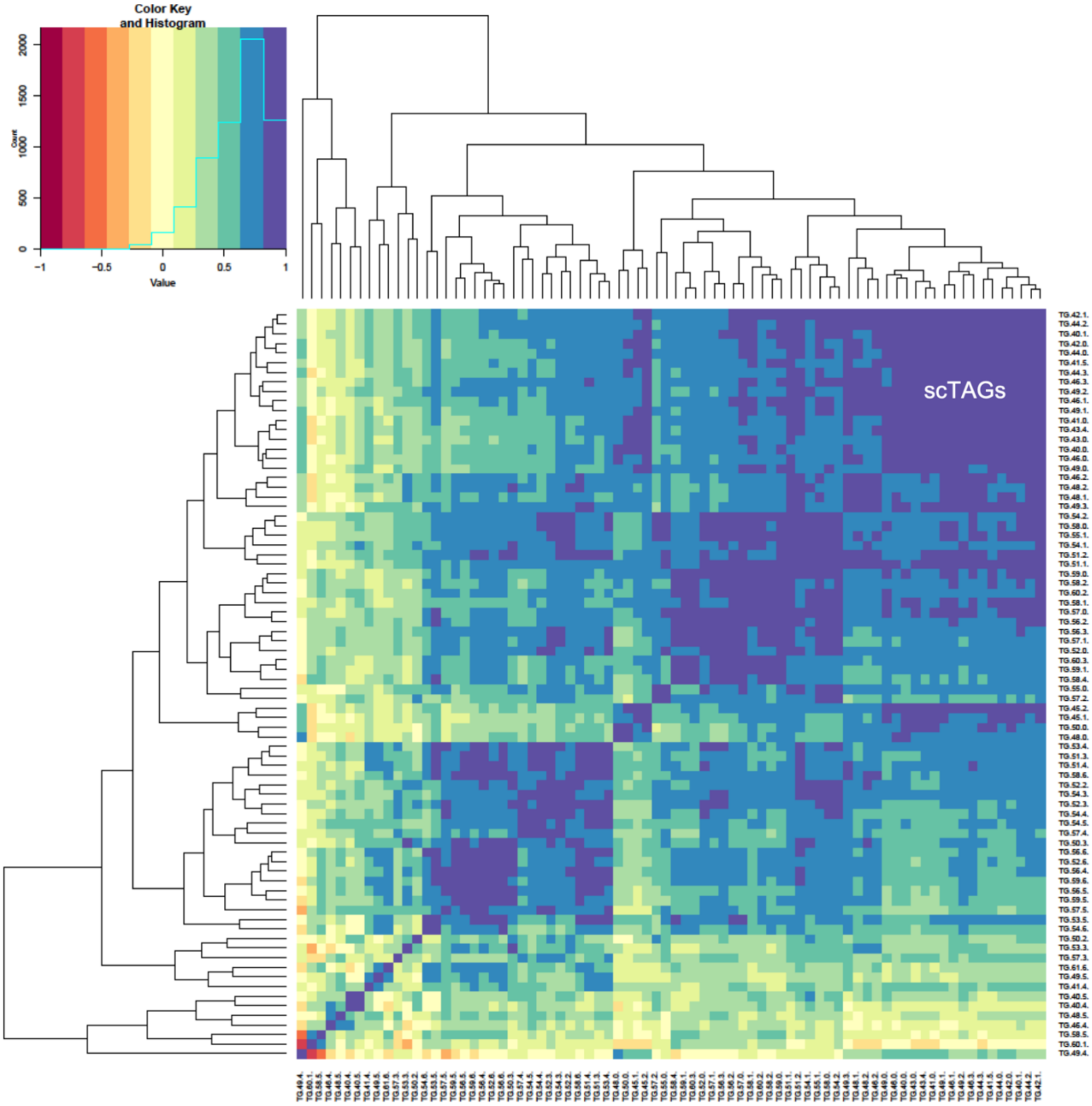

Figure 1. scTAGs form a distinct cluster. Hierarchical Cluster Analysis with Spearman correlation among the TAG profiles measured by LC-MS of all individuals after a HP meal; *n* = 9.

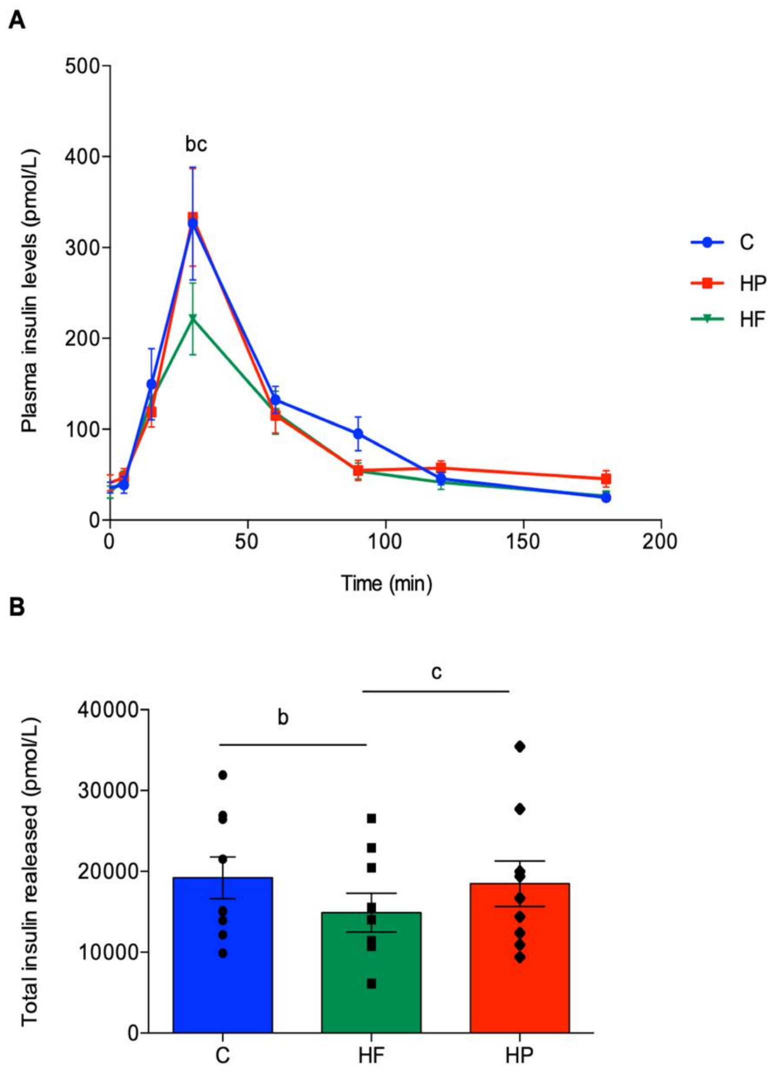

Figure 2. Plasma insulin levels. (A) Plasma insulin released after consuming a C (blue), HP (red) and a HF (green) meal. **(B)** Total plasma insulin released after consuming a C (blue), HP (red) and a HF (green) meal over 3 hours. Data are presented as mean ± SEM and analysed by two-way ANOVA with post-hoc Sidak's multiple comparisons test; a,b,c = $p \leq 0.05$, n = 9/group. a = comparisons between C and HP; b = comparisons between C and HF; C = comparisons between HP and HF **(A);** or by one-way ANOVA with post-hoc Dunnett's multiple comparisons test; * = $p \leq 0.05$, n = 9/group **(B)**.

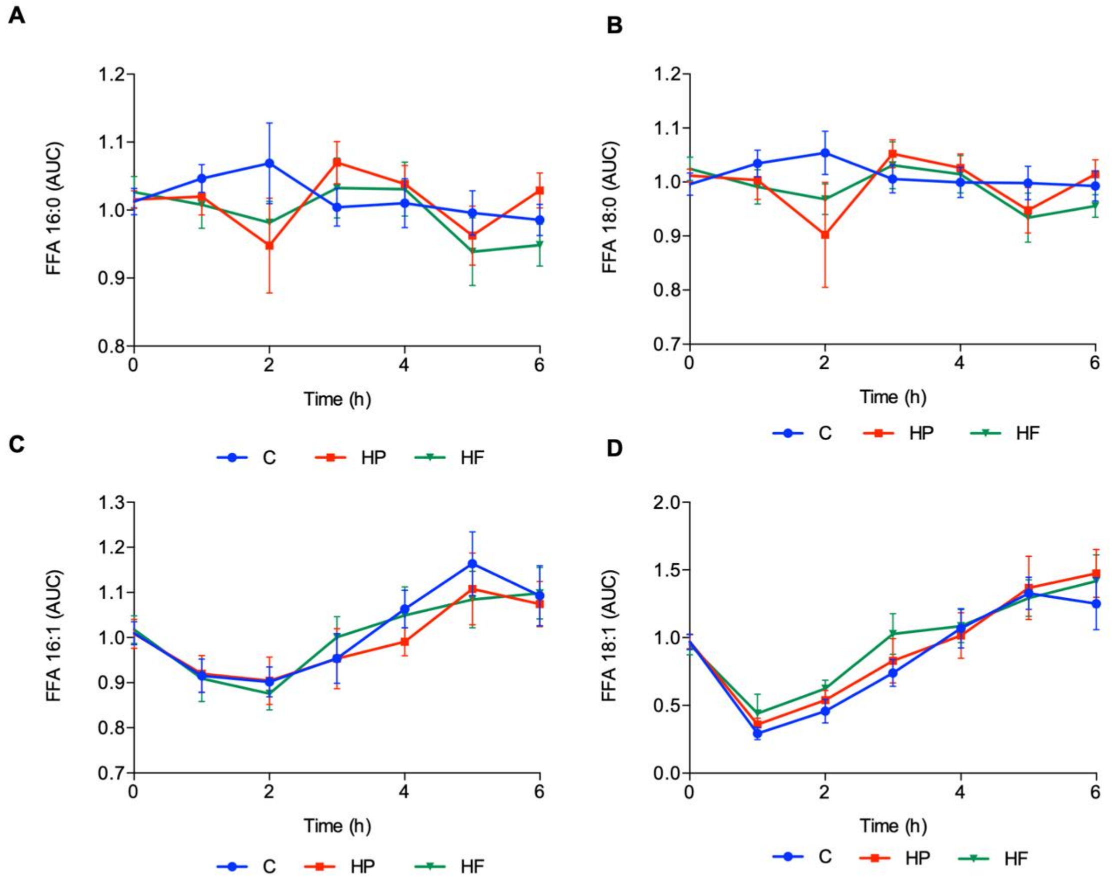

Figure 3. Plasma FFA content is not different between the groups. (A) FFA 16:0 levels measured by LC-MS over 6 hours. **(B)** FFA 18:0 levels measured by LC-MS over 6 hours. **(C)** FFA 16:1 levels measured by LC-MS over 6 hours. **(D)** FFA 18:1 levels measured by LC-MS over 6 hours. Data are presented as mean ± SEM and analysed by two-way repeated measures ANOVA with post-hoc Sidak's multiple comparisons test; * = $p \leq 0.05$, n = 9/group.

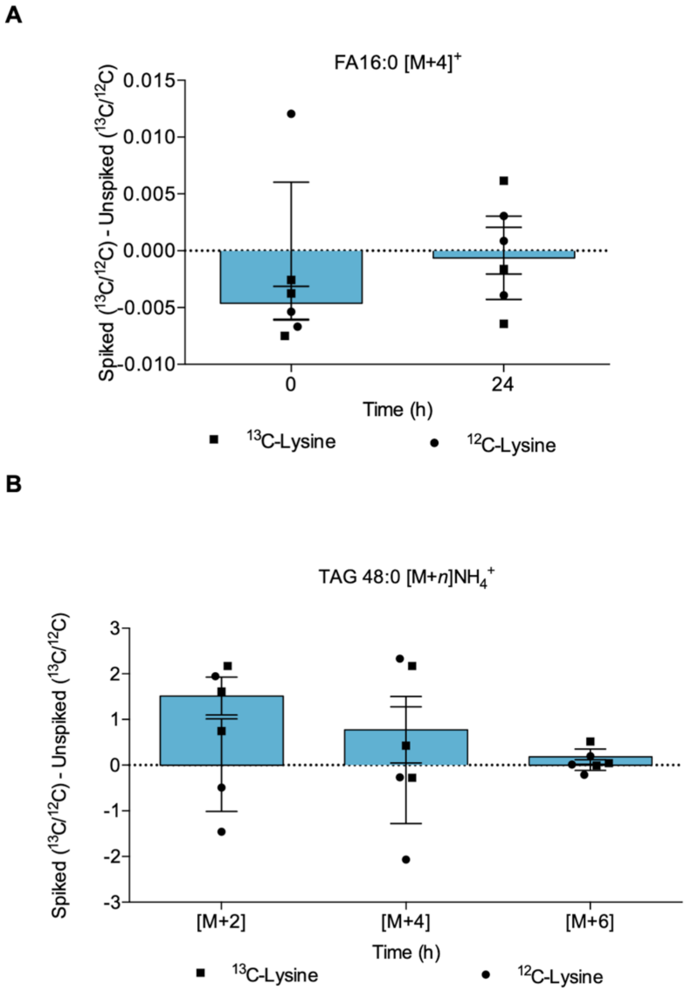

Figure 4. Carbon from $^{13}C_6$-labelled lysine is not incorporated into triacylglycerols or DNL-derived palmitate in AML 12 hepatocytes. (A) The $[M+4]^+$ ion of the picolynyl-ester of palmitate (FA16:0) detected by LC-MS was not labelled by lysine over 24 h post-supplementation. **(B)** The $[M+2]NH_4^+$, $[M+4]NH_4^+$ and $[M+6]NH_4^+$ ions of glyceryl tripalmitate (TAG 48:0) in cells detected by LC-MS were not labelled by lysine after 24 h. Data are presented as mean ± SEM and analysed by two-way ANOVA with post-hoc Sidak's multiple comparisons test; * = $p \leq 0.05$, n = 3/group.

Supplementary Table 1. Fatty acid composition of meals. The fatty acid compositions were calculated using the FoodWorks database (59), which performed a nutrient analysis of the meals.

Fatty acid (grams)	Control C	High Fat HF	High Protein HP
**FA 4:0**	0.3419	0.6838	0.3945
**FA 6:0**	0.20241	0.40482	0.23355
**FA 8:0**	0.1200527	0.237832727	0.137036364
**FA 10:0**	0.2666927	0.531112727	0.306236364
**FA 12:0**	0.2982827	0.594292727	0.342686364
**FA 13:0**	0	0	0
**FA 14:0**	1.0945009	2.154910909	1.240595455
**FA 15:0**	0.0045455	0.004545455	0.002272727
**FA 16:0**	4.994	7.763	4.3075
**FA 17:0**	0.0159091	0.015909091	0.007954545
**FA 18:0**	2.0618609	3.339630909	1.866395455
**FA 20:0**	0.0090909	0.009090909	0.004545455
**FA 22:0**	0.0113636	0.011363636	0.005681818
**FA 24:0**	0.0022727	0.002272727	0.001136364
**FA 14:1**	0.0090909	0.009090909	0.004545455
**FA 15:1**	0	0	0
**FA 16:1**	0.5338073	0.769887273	0.421263636
**FA 17:1**	0	0	0
**FA 18:1**	6.1247273	8.776727273	4.796363636
**FA 20:1**	0.0272727	0.027272727	0.013636364
**FA 22:1**	0.0022727	0.002272727	0.001136364
**FA 18:2**	1.3858873	1.624047273	0.848663636
**FA 20:2**	0	0	0
**FA 18:3**	0.1874909	0.340890909	0.194045455
**FA 18:4**	0	0	0
**FA 20:4**	0.1409091	0.140909091	0.070454545
**FA 20:5**	0.0045455	0.004545455	0.002272727
**FA 22:5**	0	0	0
**FA 22:6**	0.0363636	0.036363636	0.018181818

Supplementary Table 2. Amino acid composition of the meals. The amino acid compositions were calculated using the FoodWorks database (59), which performed a nutrient analysis of the meals.

**Amino acid (grams)**	**Control C**	**High Fat HF**	**High Protein HP**
**Alanine**	0.8576	0.8857	1.7382
**Arginine**	0.9029	0.9299	2.3174
**Aspartic acid**	1.5410	1.5921	3.8066
**Cystine**	0.3629	0.3748	0.6504
**Glutamic acid**	2.0204	2.0995	5.6847
**Glycine**	0.5189	0.5360	1.3386
**Histidine**	0.3599	0.3723	0.8495
**Isoleucine**	0.8429	0.8733	1.8245
**Leucine**	1.3075	1.3539	2.9243
**Lysine**	1.0924	1.1297	2.3081
**Methionine**	0.4874	0.5046	0.7902
**Phenylalanine**	0.8289	0.8588	1.8981
**Proline**	0.6148	0.6418	1.5943
**Serine**	1.1246	1.1597	2.1439
**Threonine**	0.7294	0.7535	1.4775
**Tryptophan**	0.1877	0.1945	0.4392
**Tyrosine**	0.6208	0.6425	1.3628
**Valine**	0.9429	0.9771	1.9829

Supplementary Table 3. scTAG masses and their corresponding inter-batch and intra-batch CVs. These TAGs are associated with the main fatty acid products from DNL including palmitate, palmitoleic acid, stearic acid and oleic acid. These TAGs have been shown to increase when DNL is raised in humans (9, 19 & 20). Samples across this study were run in two batches of roughly equal size. The technical replicates were pool samples from batch 1, the inter-batch CV was calculated as STDEV (all pools for batch X)/Mean of all pools across batch X *100%. The inter-batch CV is calculated across the two batches.

Triglyceride	***Product ion ([M+NH4]1+)***	***Intra-batch RSD/CV (%) Batch 1; Batch2***	***Inter-batch RSD/CV (%)***
TG 46:2	*792.7076*	*9.65; 6.31*	*23.6*
TG 46:1	*794.7232*	*13.1; 11.4*	*19.2*
TG 46:0	*796.7389*	*12.7; 14.5*	*15.5*
TG 48:2	*820.7389*	*12.0; 7.80*	*26.5*
TG 48:1	*822.7545*	*9.68; 9.55*	*15.9*
TG 48:0	*824.7702*	*34.3; 12.0*	*33.8*
TG 49:2	*834.7545*	*9.38; 9.25*	*22.1*
TG 49:1	*836.7702*	*12.7; 12.6*	*31.3*
TG 49:0	*838.7858*	*5.48; 12.3*	*34.0*
